# Future operation of hydropower in Europe under high renewable penetration and climate change

**DOI:** 10.1016/j.isci.2021.102999

**Published:** 2021-08-19

**Authors:** Ebbe Kyhl Gøtske, Marta Victoria

**Affiliations:** 1 Department of Mechanical and Production Engineering, Aarhus University, Aarhus, Denmark; 2 iCLIMATE Interdisciplinary Centre for Climate Change, Aarhus University, Aarhus, Denmark

**Keywords:** Energy resources, Energy policy, Energy sustainability, Energy flexibility

## Abstract

As large renewable capacities penetrate the European energy system and the climate faces significant alterations, the future operation of hydropower reservoirs might deviate from today. In this work, we first analyze the changes in hydropower operation required to balance a wind- and solar-dominated European energy system. Second, we apply runoff data obtained from combining five different global circulation models and two regional climate models to estimate future reservoir inflow at three CO_2_ emissions scenarios (RCP2.6, RCP4.5, and RCP8.5). This enables us to address the climate model uncertainty reported in previous literature. Despite large interannual and intermodel variability, significant changes are measured in the climate model signal between today and future climate. Annual inflow decreases by 31% (20%) in Southern countries and increases by 21% (14%) in Northern countries for high (mid)-emission scenarios. Projections also show impacts on seasonal profiles and more frequent and prolonged droughts in Mediterranean countries.

## Highlights

• Hydro inflow was modeled with high resolution for Europe at different CO_2_ emissions
• Climate change impact on inflow is larger than interannual and intermodel variability
• Interannual variability as well as the occurrence of extreme events are impacted
• Renewable penetration requires a higher seasonality and day-night switch of hydropower

## Graphical abstract

**Figure:**
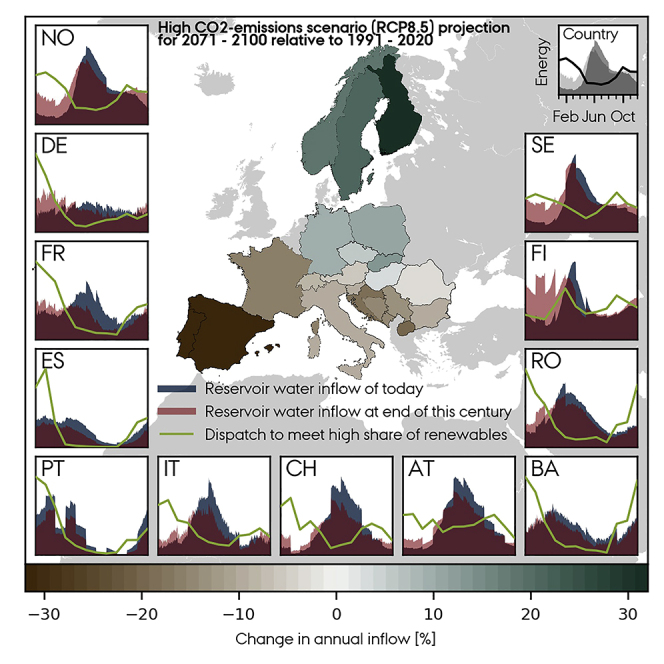

## Introduction

The power sector is facing a major transformation from using fossil fuel generators to renewable and carbon-neutral ones. The European Green Deal proposed by the European Commission (COM, 2019) envisions a climate-neutral Europe by 2050. This target could be achieved by relying on wind and solar power generation balanced by hydropower and other dispatchable generators (Victoria et al., 2020; Bogdanov et al., 2019). Hydropower is a substantial part of the European electricity generation (accounting for 16% in 2018 (IEA, 2021)) and will remain an important energy source in the future. Owing to the variable nature of wind and solar generation, their increasing penetration in power grids strengthens the need for balancing and demands a modified operation of hydropower plants (Pfeiffer et al., 2021). In some cases, the operation of hydropower to balance wind and solar could be beneficial because a seasonality similar to the natural river discharge is retained (Sterl et al., 2021).

The renewable energy supply is furthermore affected by the changing climate conditions (Cronin et al., 2018; Yalew et al., 2020; EEA, 2019). Most European regions are, at the end of the century, projected to encounter increased wind correlation lengths (Schlott et al., 2018) and higher probability of low wind regimes (Weber et al., 2018). For solar power, the radiation is projected to decrease in Northern Europe (Schlott et al., 2018; Jerez et al., 2015). Schlott et al. (2018) investigated the future European power system under climate change impacted series for solar, wind, and hydropower and found that the cost-optimal contribution of solar increases as a result of climate change. This is in agreement with the results found by (Fonseca et al., 2021) for the Southeast US. Previous studies show that hydropower is particularly sensitive to climate change (Lehner et al., 2005; Turner et al., 2017; Van Vliet et al., 2013, 2016; Schlott et al., 2018; Schaefli et al., 2007). Turner et al. (2017) applied three general circulation models (GCMs), at a low- and high-emission scenario, in a high-fidelity dam model with a variable hydraulic head to project the generation for 1,593 globally distributed hydropower reservoirs at the middle of the century. The authors selected hydropower reservoirs whose dam and turbine specifications (e.g., dam height, storage capacity, upstream catchment area, maximum turbine flow rate, etc.) are available. Van Vliet et al. (2016) used a higher number of hydropower plants (24,515) and GCMs (5) but less information (fixed hydraulic head) for each hydropower plant. Both studies, which use climate model data of daily temporal and 0.5 ° × 0.5 ° (≈50km×50km) horizontal resolution, project a north-south European division in which the Nordic countries gain an increase in annual hydropower production whereas the Mediterranean and Balkan countries suffer from a reduction. However, the two studies do not agree on the outlook for global hydropower. The former projects a change in annual electricity production of ±5% depending on the GCM, whereas the latter projects a robust (across all GCMs) decrease in the range from 0.4% to 6.1%. Because projections of hydropower impacts at a global and regional scale are known to vary (Yalew et al., 2020; Cronin et al., 2018), climate model uncertainty has been identified as a significant research gap (Cronin et al., 2018). Moreover, both the aforementioned analyses focus on investigating the impact of climate change on annual figures and assume that the hydropower operation is independent of the rest of the power systems.

The available energy for electricity production at a hydropower plant is determined by the local characteristics such as topography, snow formation, temperature, and precipitation which might not be adequately represented in the GCM owing to its coarse horizontal resolution. By downscaling the climate model simulation results, using a regional climate model (RCM), the local and regional characteristics obtain a higher representativeness (Christensen and Kjellström, 2020). A comprehensive comparison of RCMs for Europe was conducted by Christensen and Kjellström (2020) in which a 5 × 5 GCM-RCM matrix was analyzed for temperature, wind speed, and precipitation. It showed that the climate model signal was generally more influenced by the choice of GCM than RCM but that the RCM has higher influence in mountainous regions owing to the added surface detail. Downscaling itself introduces new sources of uncertainty, comparable with that of GCM uncertainty in magnitudes (Suzuki-Parker et al. (2018)), which in particular applies to precipitation (Guillod et al., 2017). They can, however, be limited by considering the full ensemble instead of a particular RCM (Wooten et al. 2017).

**Figure 1:**
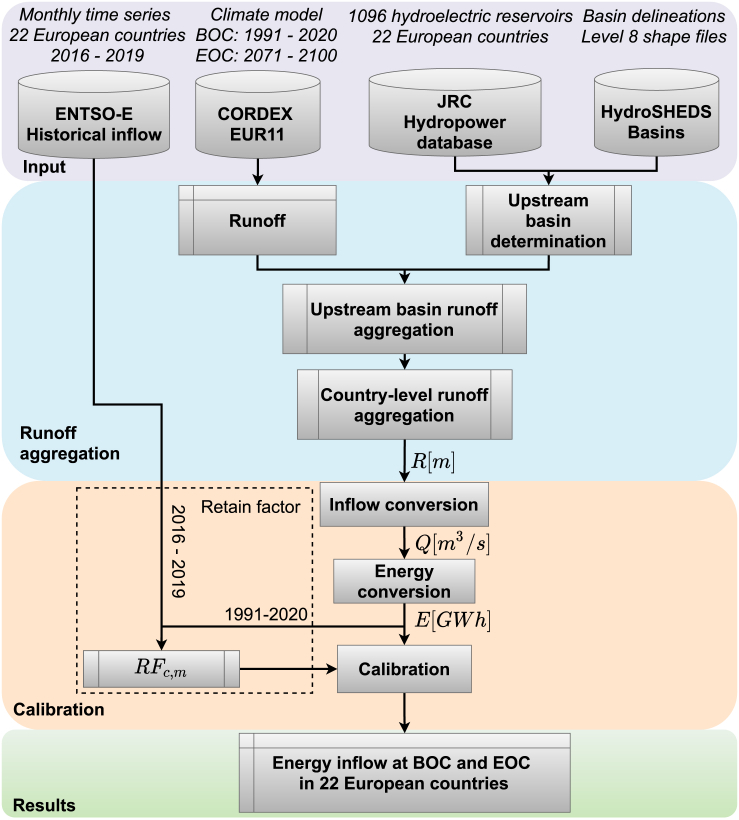
Workflow to obtain hourly resolved inflow time series at the two periods, beginning of the century (BOC), from 1991 to 2020, and end of the century (EOC), from 2071 to 2100

In this study, we investigate how future European hydropower is required to operate when accounting for (1) the increasing penetration of renewable generators in the energy system and (2) the climate change impact on the available hydropower resource. The former is based on the 2050 decarbonized cost-optimal European energy system (Victoria et al., 2020), and the latter is based on climate model data acquired from the Coordinated Regional Climate Downscaling Experiment (CORDEX) (Giorgi et al., 2009; WCRP, 2020). We combine the two aspects and investigate whether the operation needed to meet the mixed effect of changed balancing requirements and climate change is achievable. Furthermore, our approach includes two significant novelties. First, our analysis is limited to evaluating changes not only on the annual resource but also on the seasonal and daily availability, which enables us to investigate how hydropower is influenced by the frequency and duration of droughts. Second, we apply a more extensive climate model ensemble with increased spatial resolution of each ensemble member than prior studies. We focus on runoff because the inflow in reservoirs is highly correlated to the runoff within the upstream basins of the reservoir (Liu et al., 2019). Furthermore, we use an advanced conversion scheme to model inflow from climate model runoff data in European reservoirs. To our knowledge, such comprehensive analysis of a large runoff-climate model ensemble with an advanced inflow conversion scheme has not been conducted before. An emphasis is furthermore put on the variability caused by the differences between the climate models (intermodel variability) and within the considered period (interannual variability) to quantify the robustness and significance of the resulting projection. The time series that we produce in this project are released under an open license. They include hourly-resolved time series of inflow for 22 European countries at the beginning of the century (BOC) from 1991 to 2020 and the end of the century (EOC) from 2071 to 2100, under three CO_2_ emissions scenarios (RCP2.6, RCP4.5, and RCP8.5), for ten different GCM-RCM climate model combinations, as well as the average of the model ensemble. See the workflow in Figure 1, and the full methodology description in STAR Methods.

The decarbonized energy system for 2050 in the study by Victoria et al. (2020) was obtained with the *PyPSA-Eur-Sec* model which optimizes the capacity and dispatch of every generation, storage, and transmission technology, with the total annualized system cost as the objective function, subject to a global CO_2_ constraint. The model assumes perfect competition and foresight as well as long-term market equilibrium. The system, including the electricity and heating sectors, is optimized every 5 years from 2020 to 2050 following a myopic approach, i.e., in every optimization step, only information regarding that year is available. Technologies installed in previous time steps remain in the model until they reach the end of their lifetime. The CO_2_ constraints in every time step correspond to a carbon budget of 21GtCO_2_ which enables avoiding human-induced warming above 1.75°C with a probability of >66%, assuming current sectoral distribution for Europe, and equity sharing principle among regions. In PyPSA-Eur-Sec, reservoir hydropower in every country is defined as a storage unit with a certain discharge capacity and time. The inflow time series is used to charge the storage, and with this, the energy system model plans the optimal dispatch that minimizes the total system cost, based on the available renewable generation and electricity and heating demand in every time step. The model uses a single year of historical weather data, as well as historical heat and electricity demand, as input. The *Baseline - Early and Steady pathway* scenario in the study by Victoria et al. (2020) will in this study work as a benchmark of the decarbonized European energy system. The technology costs are not altered.

## Results and discussion

### Changes in the hydropower operation as wind and solar penetration increases

**Figure 2:**
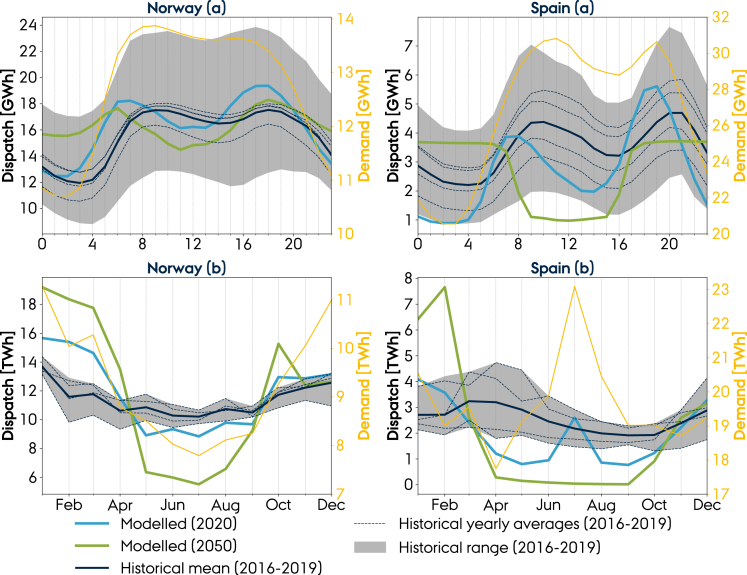
Modeled and observed (A) intraday and (B) seasonal operation of hydropower plants The shaded gray area indicates the range of the historical observations. The historical observations are based on hourly reservoir and run-of-river hydropower generation from ENTSO-E. For plot distinguishing between the individual historical years, see Figure S9. Similar plots for other countries are shown in Figure S10.

For the sake of clarity, we focus the discussion on hydropower in Norway and Spain as countries representative of Northern Europe and Mediterranean climates and which are projected to experience contrasting impacts of the decarbonization of the energy system (Victoria et al., 2020) and of climate change on hydro resources (Van Vliet et al., 2016; Turner et al., 2017). Figures in Supplemental Information extend the analysis to other countries. Figure 2 presents the intraday and seasonal profile of the hydroelectricity from historical observations and the cost-optimized model of the European energy system in 2020 and 2050 from Victoria et al. (2020). Because the energy system model in the mentioned study is based on a single year of weather data, this subsection analyses solely the effect of the energy system decarbonization on hydropower, while the climate variable influence is omitted. The modeled intraday operation in 2020 is within the historical range in both countries and captures the morning and afternoon peaks caused by the load curve. The modeled intraday generation in 2050 shows a minimal change in the profile for Norway, whereas Spain shifts from matching the load curve in 2020 to showing a binary operation. The day-night switch can be explained by the large installation of solar capacity in Spain which provides high generation at day but requires balancing at night.

The modeled hydropower does not resemble the historical seasonal dispatch as well as the intraday profiles. In 2020, modeled dispatch in both countries shows a lower magnitude during summer and higher during winter, relative to the historical observations. In addition, Spain shows a hydro generation peak in July caused by high electricity demand. The model assumes perfect foresight (i.e., the weather and inflow are known for the entire year) which might cause too ideal operation of hydropower. By 2050, the high wind and solar penetration forces the energy system to rely more heavily on hydropower during winter. The Spanish hydropower is particularly influenced by the solar penetration, showing almost zero dispatch from April to September, and a short period during winter with a high level of dispatch. Victoria et al. (2020) showed that countries surrounding Norway relied heavily on wind, whereas the ones surrounding Spain had solar as their main energy supply. The Norwegian hydropower could thus be treated as the battery of European wind power, and hydropower in Spain of solar. This distinction is, however, not clearly shown in the temporal characteristics of the future dispatch in Norway and Spain. Norwegian dispatch in 2050 is higher in winter, in which wind generation is at its maximum, and thus appears to be affected more by the high solar penetration at the European level.

**Figure 3:**
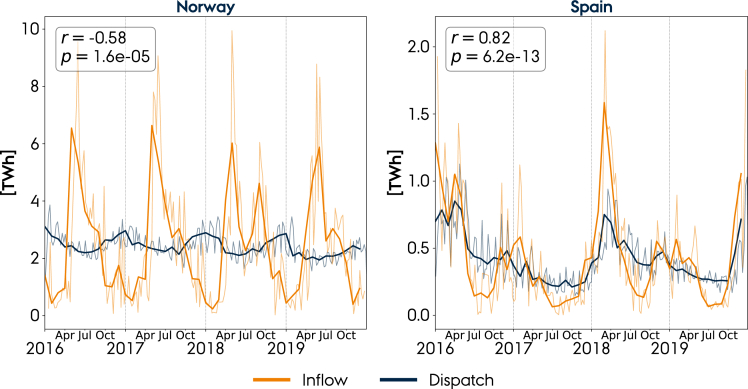
Observed hydropower reservoir inflow and dispatch from 2016 to 2019 from ENTSO-E, with *r* indicating the inflow-dispatch correlation and *p* the statistical significance Thin lines indicate weekly values, and thick lines monthly averaged. Similar plots for other countries are shown in Figure S11.

Figure 3 illustrates the historical reservoir water inflow and hydropower dispatch, which are negatively correlated in Norway. The inflow in Norway has a summer peak in June caused by the melting of snow, whereas the dispatch peaks in winter; thus, a high share of the hydro resources are saved for five months. Conversely, Spain does not show such a capability of shifting the seasonal dispatch based on the historically strong inflow-dispatch correlation. The inflow in Spain, influenced by rain patterns but not by deicing, peaks in winter, while the electricity demand is higher in summer. The strong inflow-dispatch correlation in Spain can thus not be explained by the electricity demand curve, but instead by the lack of seasonal storage capacity. This is also illustrated in Figure S11 in which the inflow-dispatch correlation for nine countries shows a strong negative correlation with the country-average size of the reservoirs (ratio between the energy capacity and power capacity of hydropower reservoirs). The increased seasonality in the hydropower operation suggested by the model seems, from a historical perspective, feasible in both Norway and Spain for different reasons. Norway can meet the seasonally shifted hydropower operation due to large reservoirs, whereas Spain is able to comply with increased hydropower generation in winter because it is concurrent with the peak inflow. Figure S10 shows the intraday and seasonal operation for the remaining countries. For Austria and Romania, a similarly increased seasonality is shown in the hydropower operation required by the model. However, the two countries are subject to a peak inflow in summer, and because the hydropower generation in those countries is correlated with the inflow, the modeled operation in 2050 is questionable.

The required change in operation may contribute to higher ramp rates, see Figure S12 (although extreme ramps show low probability), which can lead to a reduced lifetime of the hydropower plants due to higher mechanical stresses (Bakken and Bjorkvoll, 2002). This is consistent with previous studies (Pfeiffer et al., 2021; Gallego-Castillo and Victoria, 2021) which showed the same effect when only increasing the wind power capacity within a US region. Future operation of hydropower also needs to consider that the artificial flow fluctuations downstream of reservoirs caused by hydropeaking might entail negative consequences for aquatic organisms (Schmutz and Sendzimir, 2018).

### Changes in hydropower inflow caused by climate change

As a prelude to this section, it is noteworthy that we are already witnessing climate change impact on hydropower resources. Figure S18 presents simulated Norwegian reservoir inflow, based on historical precipitation and temperature measurements, from 1958 to 2017, produced by Holmqvist (2017), and historical annual mean temperature in Norway acquired from climate reanalysis (Hersbach et al., 2018). This is a representation of the historical climatic evolvement (energy capacity is assumed constant in the simulation by Holmqvist) and shows a clear increasing trend in temperature and annual inflow.

**Figure 4:**
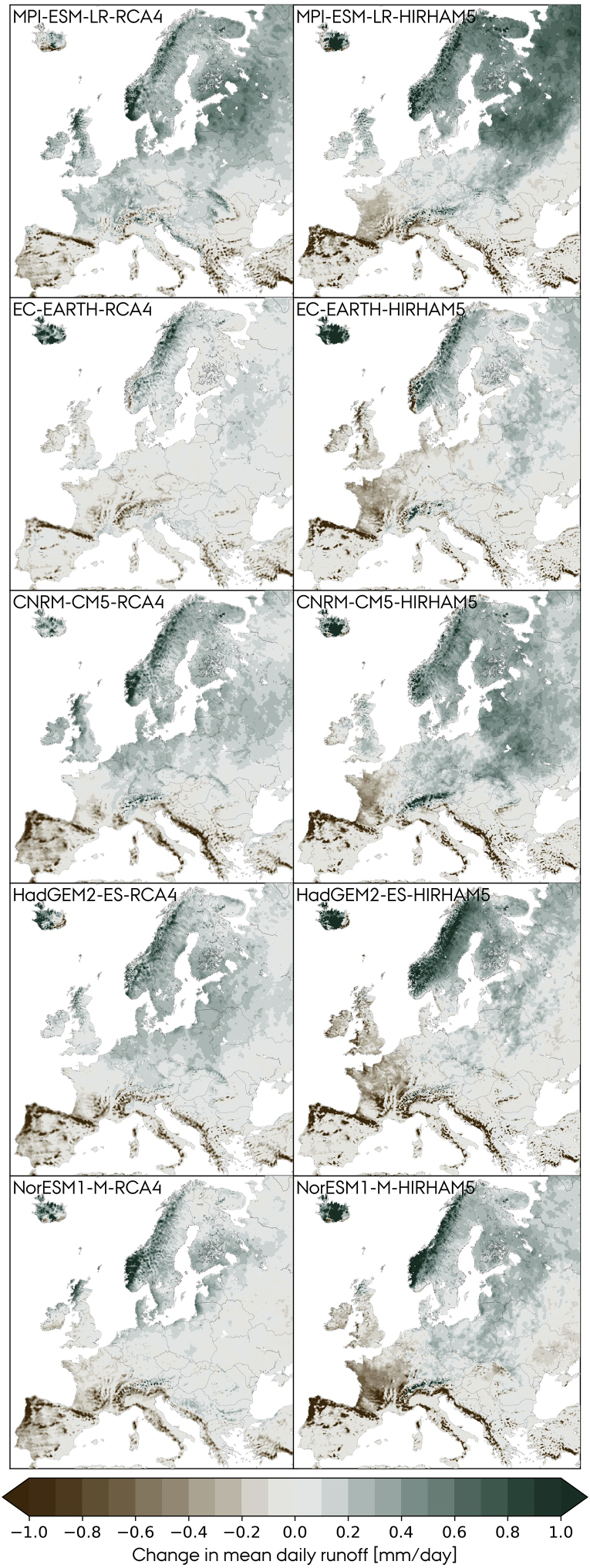
Change in the mean daily runoff [mm/day] at the EOC period relative to the BOC for the RCP8.5 scenario

**Figure 5:**
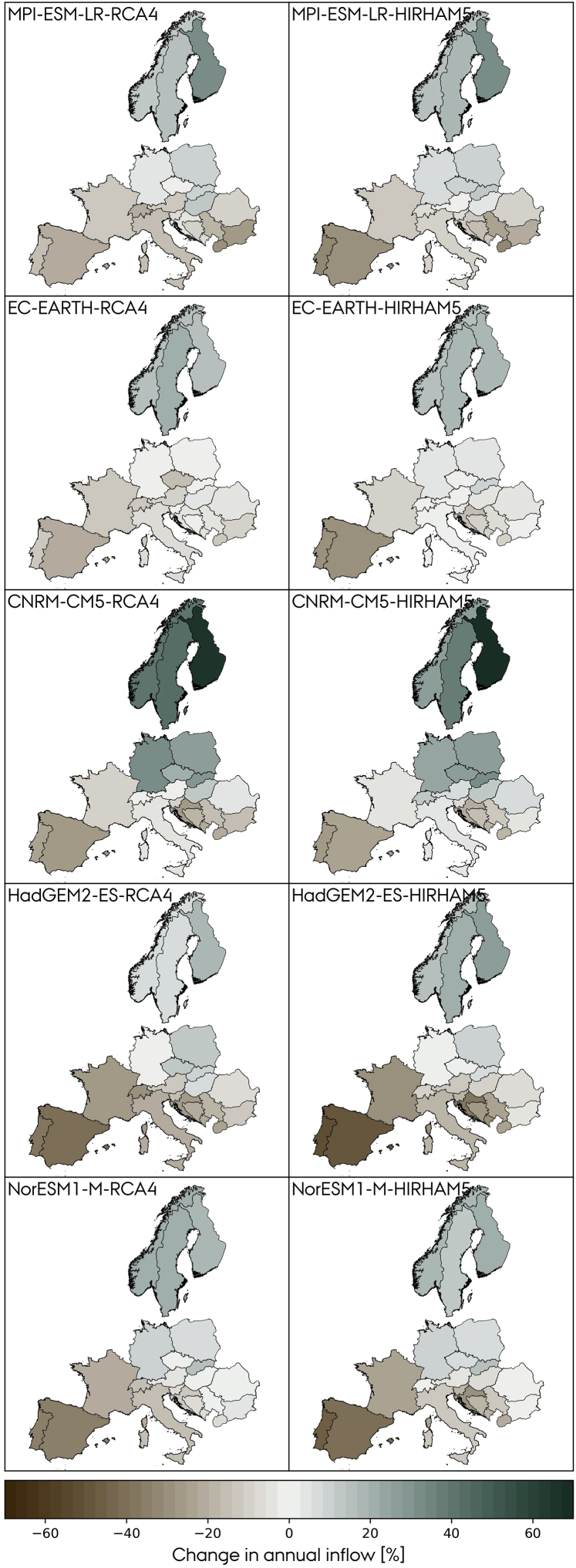
Relative changes in annual country-aggregated inflow at the EOC period relative to the BOC for the RCP8.5 scenario

As a preliminary step, we evaluate the absolute change in mean daily runoff at the EOC period relative to the BOC (Equation 12). Figure 4 shows the results for the combinations of 5 GCMs and 2 RCMs for the RCP8.5 scenario. The signal is consistent for the outer areas of the domain, i.e., runoff increases in northern Europe and decreases in the south. The models vary in the magnitude and direction of change in some regions. France is a good example of the latter: Runoff increases or shows negligible changes in combinations using RCA4 as RCM but decreases when HIRHAM5 is used. The corresponding matrix of relative change in annual inflow (Equation 13) derived from the climate model runoff is presented in Figure 5. Consistently across models, for the RCP8.5 scenario, Mediterranean countries show a significant reduction at the EOC period while Nordic countries show a significant increase. When comparing the runoff and inflow matrices, some considerations must be taken. First, the inflow matrix is based on relative changes, i.e., countries with low hydropower capacities are more sensitive to absolute changes in runoff, which explains why, e.g., Finland in some models shows the largest relative inflow increase when it is not reflected on the runoff. Second, the modeled inflow is based on the location of the hydropower plants, see Figure S2, and e.g., France has most of its plants located in the south, so the inflow is barely affected by the runoff change in the north.

**Figure 6:**
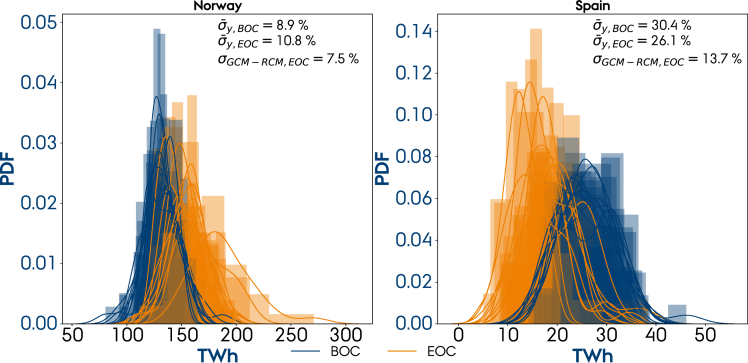
Kernel density estimations of the probability density functions for the annual inflow in Norway and Spain at the BOC (blue) and EOC (yellow) periods for the RCP8.5 scenario with the corresponding interannual variability, ${\bar{\sigma }}_{y,\text{BOC}}$ and ${\bar{\sigma }}_{y,\text{EOC}}$, and the intermodel variability at the EOC, $\sigma_{\text{GCM}-\text{RCM},\text{EOC}}$

We now focus on analyzing the intermodel and interannual variability. Figure 6 depicts the kernel density estimations of the probability density functions of the annual inflow in Norway and Spain at the BOC and EOC 30-year periods for the ten different climate models. As Figure 5 anticipates, the climate models all agree on an increase in annual inflow in Norway and a reduction in Spain. The interannual variability, averaged for the ten climate models, ${\bar{\sigma }}_{y}$, is projected to increase from 8.9% (of the mean annual inflow) at the BOC period to 10.8% at the EOC in Norway and decrease from 30.4% to 26.1% in Spain. The variability between the ten climate model (mean) projections, i.e., the intermodel variability $\sigma_{\text{GCM}-\text{RCM}}$, is 7.5% for Norway and 13.7% for Spain at the EOC period (the intermodel variability at the BOC is zero owing to calibration with observed inflow). According to analysis of variance (ANOVA) (Weiss, 2005), because the variability between the ten climate model projections, $\sigma_{\text{GCM}-\text{RCM}}$, is smaller than the variability within, ${\bar{\sigma }}_{y}$, we can treat the ten climate model projections at the EOC as being from the same population. Based on that result, we group the ten climate model projections into one ensemble consisting of 30 (years) x 10 (climate models) observations. The variability contributed by changing the GCM or RCM is examined in Figure S17. It is shown that the range of the mean annual inflow ${\bar{E}}_{EOC}$ and the interannual variability ${\bar{\sigma }}_{y,EOC}$ is generally larger when varying the GCM than RCM. This result could indicate that the intermodel variability is mainly due to the variation in the GCM, consistent with Christensen and Kjellström (2020). However, to verify this indication, a larger number of RCMs would be needed.

**Figure 7:**
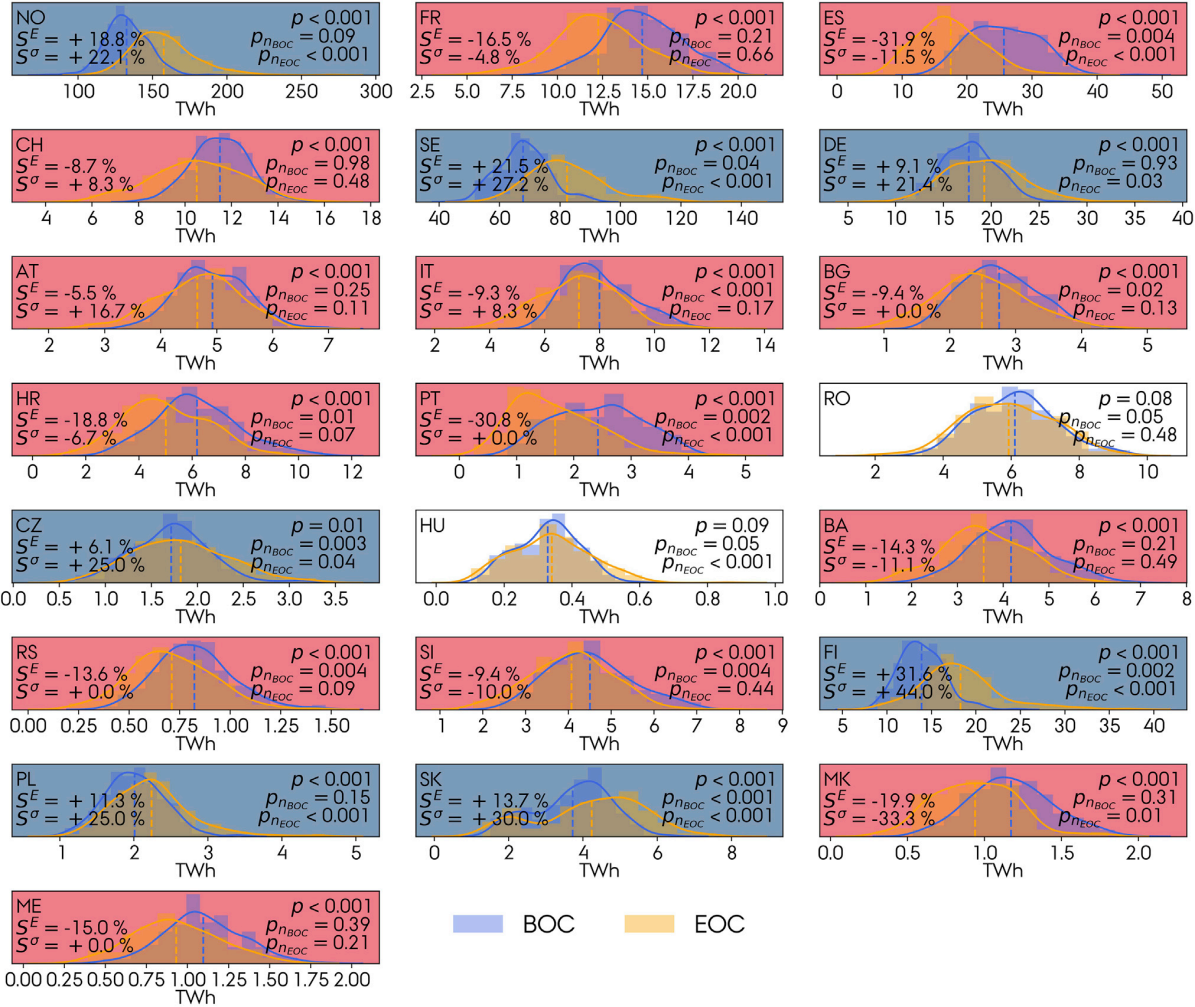
Ensemble distributions of the annual inflow at the BOC and EOC 30-year periods for the RCP8.5 scenario Dashed lines indicate the mean values of the distributions. The sets are normally distributed if $p_{n}>0.05$ based on the Shapiro-Wilk test. For the countries with a statistically significant change (p<0.05), a blue (red) shade indicates an increase (decrease) in the annual inflow. $S^{E}$ and $S^{\sigma }$ correspond to the relative change in annual inflow and interannual variability caused by climate change. For RCP4.5, see Figure S13.

Figure 7 shows the distributions of the annual inflow from the climate model ensemble at the BOC and EOC periods (each consisting of 300 observations) for the 22 countries under analysis and the RCP8.5 scenario. For France, Switzerland, Austria, Bosnia and Herzegovina, and Montenegro, the Shapiro-Wilk test indicates that the data at both eras follow a normal distribution ($p_{n}<0.05$). For the remaining countries, because all sets show similar (bell-shaped) normal distribution characteristics based on the kernel density estimations, in spite of the result from the Shapiro-Wilk test, we proceed to the *t*-test. The null hypothesis stating that the inflow at EOC and BOC are equal can be rejected for all countries except Romania and Hungary. Figure 7 also shows the relative change in annual inflow $S^{E}$ and mean interannual variability $S^{\sigma_{y}}$ (normalized with the mean annual inflow). The latter is calculated for each model and averaged as in Figure 6, so $S^{\sigma }$ does not represent the width of the distributions plotted in Figure 7 because they also include the intermodel variability. However, they might be similar because we showed that the interannual variability is larger than the intermodel variability. Countries in which annual inflow increases also show an enlarged interannual variability, that is, countries that are expected to benefit from more abundant hydropower resources at the EOC will also suffer from stronger year-to-year fluctuations. Countries experiencing a reduction in annual inflow do not show the same unanimity regarding the direction of change of the interannual variability: The latter increases for countries in central Europe (Switzerland, Austria, and Italy) but decreases in Mediterranean countries (France, Spain, Croatia, Bosnia and Herzegovina, Slovenia, and North Macedonia).

**Figure 8:**
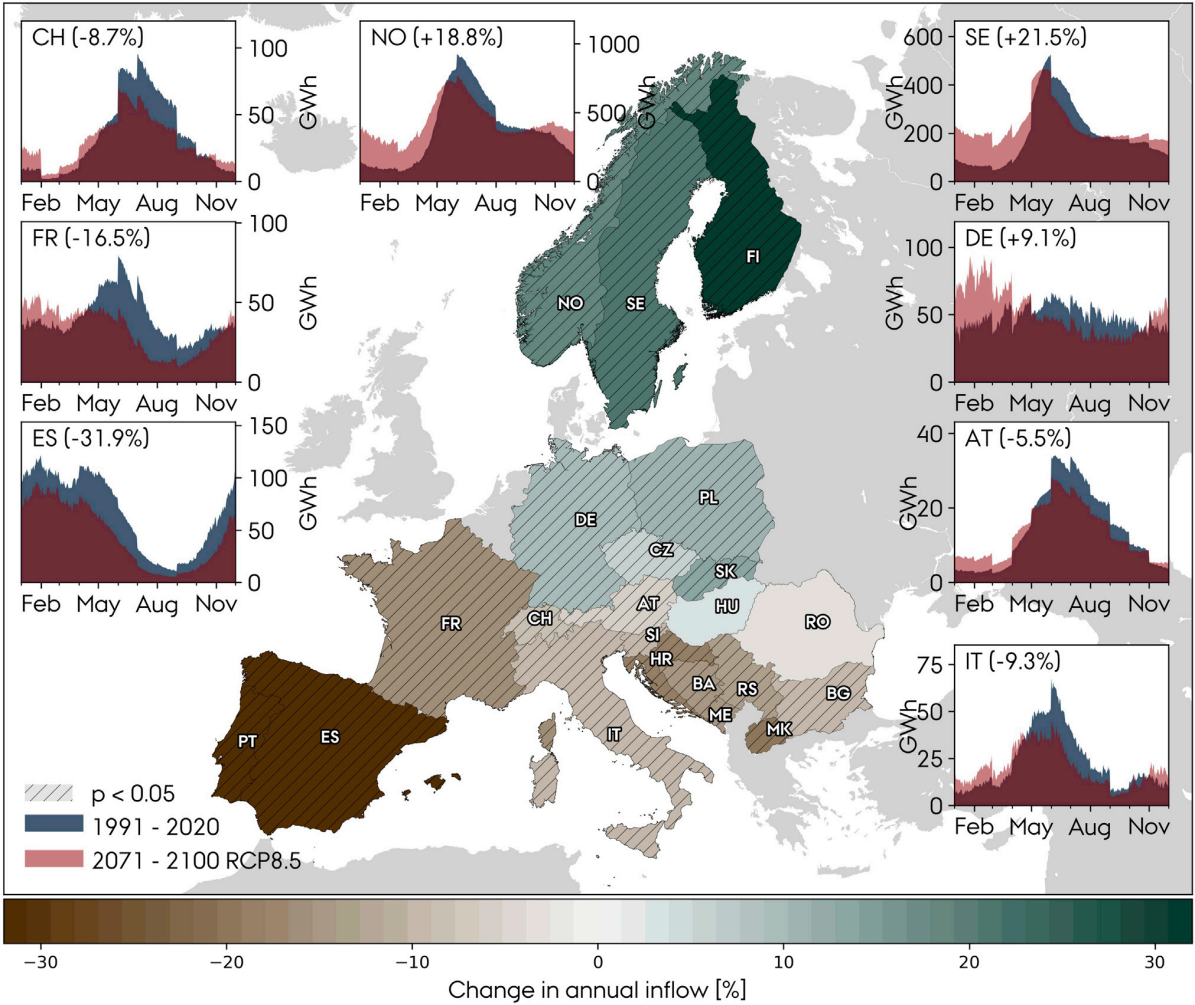
Ensemble mean relative change in annual inflow and change in seasonal inflow profile for RCP8.5 Dashed patterns indicate results that are statistically significant (p<0.05). For RCP4.5, see Figure S14.

The average of the signal from the ten climate models, i.e., the ensemble mean, is presented in Figure 8 in which the relative change in the annual inflow is indicated on the map of Europe, and the seasonal inflow profiles, of daily resolution, for the two eras are given for the countries with the largest hydropower capacities. The consequence of climate change is latitude-dependent illustrated by the Northern European countries facing an increase in the annual inflow, while the Southern European countries, e.g., France, Austria, Switzerland, and Italy, all encounter a scarcer annual resource available for hydropower production. The most extreme impacts are found for Spain and Portugal, 30% decrease in annual inflow under the RCP8.5 scenario, the Balkan countries (10–20% decrease) and the Nordic countries (20–30% increase). All countries except Spain are projected to encounter increased available hydro resources during the winter because less water is stored as ice at high altitudes, but it decreases during spring and summer. Spain does not show any change in the shape of the seasonal profile, but it suffers from a reduced inflow throughout the year.

**Figure 9:**
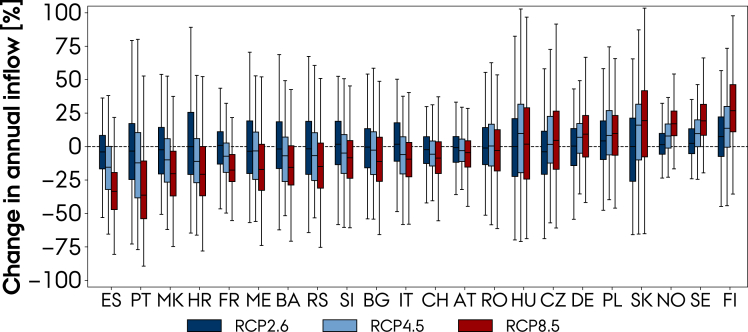
Box and whisker plot of changes in the annual inflow at the EOC period (2071–2100) relative to the BOC (1991–2020) for 22 European countries at the three emission scenarios

The RCP8.5 scenario has a low probability, given the current emission trends (Hausfather and Peters, 2020), but can work as an upper bound of the future climate change impact. The RCP4.5 scenario is, on the other hand, considered a likely scenario with current mitigation policies (Hausfather and Peters, 2020). Figure 9 presents the box and whisker plots of the relative change in annual inflow for the 22 countries at the three emission scenarios (each plot represents the distribution of 6 × 30, 8 × 30, and 10 × 30 model years for RCP2.6, RCP4.5, and RCP8.5). When looking at the median (and other percentiles shown by the box and whiskers plot), the results are generally consistent in the direction of change across the scenarios. For the RCP4.5 scenario, Spain and Portugal still show a robust 11–14% decrease in the annual inflow at the EOC period, the Balkan countries (except Romania) show a 3–10% decrease, while the Nordic countries show an 8–14% increase. For Romania, the impact diverges between a 1% increase and 3% reduction across the RCP4.5 and RCP8.5 scenarios.

**Figure 10:**
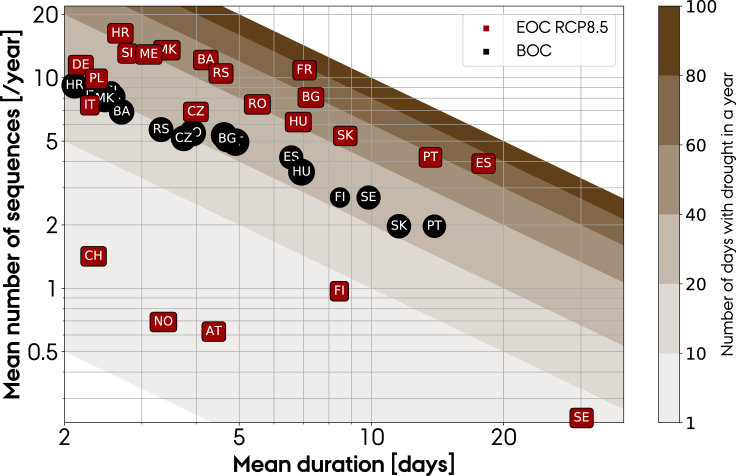
Duration and frequency of droughts, determined as consecutive days with inflow less than the 10^th^ percentile of inflow at the BOC, evaluated for the BOC and EOC periods under the RCP8.5 scenario For RCP2.6 and RCP4.5, see Figure S15, and for periods with overflow, see Figure S16.

Furthermore, we look at the frequency and severity of extreme events in inflow and how they are impacted by climate change. We define droughts as consecutive days with inflow <10^th^ and overflow periods when inflow >90^th^ percentile. Figure 10 shows the frequency and duration of droughts in the different countries under the RCP8.5 scenario (see Figure S15 for droughts under the RCP2.6 and RCP4.5 scenarios). Similarly, overflow periods are shown in Figure S16. As hydropower is expected to balance wind and solar fluctuations, severe droughts lasting several weeks could stress the system operation. Unfortunately, for most countries, climate change is expected to increase the length of drought periods and their frequency. The observed tendency is stronger for Mediterranean countries. In addition, less frequent and shorter periods of overflow are expected in all countries, except Poland, Germany, Czech Republic, Finland, and Slovakia.

As a final step, we investigate the impacts that the changes in inflow would have on the coupled electricity and heating European energy system. To that end, we compare the cost-optimal net-zero emission system in 2050, when including the climate projected hydro inflow time series at RCP8.5 (ensemble mean) at the BOC and EOC periods. For those countries where significant changes in hydro resources at the EOC period are expected, we find that the optimal wind and solar capacities vary to compensate them (see Figure S19). However, the temporal patterns of hydro dispatch remain almost identical. The mean daily and seasonal patterns are imposed by the need to balance wind and solar, while the latter is enabled by the large reservoir capacity in most countries.

### Limitations of the study

In this section, we review the main limitations of our analysis. We assume that the population and electricity consumption in every country do not change toward the future. We neglect the possible alteration in operation of hydropower owing to flood control or changes in water supply patterns. In PyPSA-Eur-Sec, perfect foresight for the entire year is assumed. On a daily basis, this assumption does not have a strong impact, but on a seasonal basis, the hydropower operation becomes too ideal as a consequence of this. Furthermore, future technology costs are subject to considerable uncertainties that could have an influence on the cost optimization of the dispatch and capacity layout of the energy system. In this study, the evolution of cost assumptions for different technologies is exogenously fixed. In addition, the energy system model includes heating, but electrification of other sectors, e.g., transportation and industry, and the flexibility provided by them, might change the operation as well. In future operation, ramp values larger than historical values appear but they show extremely low probability. This is also a consequence of unconstrained ramping in our model. In the inflow conversion model, calibration with ENTSO-E observations is based on a time range of four years. Inflow has a large interannual variability which might not be sufficiently captured in this short period. The accuracy of our model is thus limited by the representativeness of the available observations.

### Conclusion

In this work, we have investigated the impacts of climate change and high penetration of wind and solar power in the operation of reservoir hydropower plants in Europe. Because hydropower constitutes a reliant, stable, and carbon-neutral energy source and represents a significant share of the European power generation, this combined effect is important to improve the basis of planning the future energy system. The novelty in our approach exists in the inflow conversion scheme based on data from a large climate model ensemble of high spatial and temporal resolution.

First, by analyzing the cost-optimal hydropower dispatch from a fully renewable energy system, we showed that to balance the renewable generation, an increased seasonality of hydropower operation is required. With the expected large solar capacities in Southern Europe, hydropower plants located in this region are required to mostly operate during winter and nighttime. The perfect foresight, typically assumed in energy models, implies an optimal use of the hydropower balancing potential throughout the year. For most countries, the new dispatch patterns seem possible, based on the historical operation. However, for countries with low reservoir energy capacity and summer inflow peaks, e.g., Austria, Switzerland, and Italy, the hydropower dispatch shift from summer to winter, included in the model, may not be feasible.

Second, we showed that despite large interannual and significant intermodel variations, we can still detect a change in the inflow caused by climate change. By locating the upstream basins that hydropower stations were located in, runoff from ten regional climate models under three different emission scenarios was converted into inflow time series for the EOC period, ranging from 2071 to 2100. For the high emissions scenario (RCP8.5), using a paired t-test, we found a statistically significant change in annual inflow at the EOC period, relative to BOC, for 20 out of 22 European countries under analysis. The ensemble mean relative change in annual inflow is projected to decrease in Mediterranean and Balkan countries. Spain and Portugal face the largest reduction: 31–32% for the high-emission scenario and 11–14% for the mid (RCP4.5). Balkan countries (except Romania) face a reduction in the range of 9–20% (3–10%) for the high (mid)-emission scenario. On top of that, both regions will suffer from more frequent and prolonged droughts. Conversely, the ensemble mean relative change in annual inflow is projected to increase in Northern Europe. Annual inflow in Nordic countries increases in the range of 19–32% (8–14%) for the high (mid)-emission scenario. The frequency of overflow events is expected to increase for Finland but not for Sweden and Norway. The climate projections show that climate change affect the interannual variability as well. Nordic countries, Germany, Poland, and Slovakia robustly show a larger interannual variability under both mid and high emissions, whereas for Switzerland, Italy, Spain, Bosnia and Herzegovina, and Croatia, it is reduced. Climate change impact on hydro resources will require additional wind and solar power capacities in southern countries.

## STAR★Methods

### Key resources table

**Table:** 

REAGENT or RESOURCE	SOURCE	IDENTIFIER
**Deposited data**		

Modeled dispatch	Victoria et al. (2020)	https://doi.org/10.1038/s41467-020-20015-4
Historical dispatch for Austria, Bulgaria, France, Italy, Portugal, Romania, Switzerland, Montenegro, Norway, Spain, and Sweden from 2016 to 2019	ENTSO-E Transparency Platform	https://transparency.entsoe.eu/
Historical reservoir filling level for Austria, Bulgaria, France, Italy, Portugal, Romania, Switzerland, Montenegro, Norway, Spain, and Sweden from 2016 to 2019	ENTSO-E Transparency Platform	https://transparency.entsoe.eu/
Historical inflow for Norway from 1958 to 2017	Holmqvist (2017), NVE	https://www.nve.no/
Historical inflow for Sweden from 1980 to 2019	Swedenergy	https://www.energiforetagen.se/
Historical inflow for Spain from 1990 to 2019	Red Eléctrica De España	https://www.ree.es/es/datos/publicaciones/series-estadisticas-nacionales
Historical inflow for Bosnia Herzegovina, Croatia, Czech Republic, Finland, Germany, Poland, Serbia, Slovakia, Slovenia, Hungary, and North Macedonia	Wattsight	https://wattsight.com
Climate model data	WCRP CORDEX	https://esgf-data.dkrz.de/search/cordex-dkrz/
Locations of hydropower plants	JRC	https://data.jrc.ec.europa.eu/dataset/52b00441-d3e0-44e0-8281-fda86a63546d
Basin delineation polygon files	HydroSHEDS	https://hydrosheds.org/page/hydrobasins
Climate model projected inflow, data	This paper	https://doi.org/10.5281/zenodo.5106349
Clima model projected inflow, code	This paper	https://doi.org/10.5281/zenodo.5109492

**Software and algorithms**		

Python v3.7.10	Python Software Foundation	https://www.python.org/
ATLITE v0.0.2	Hofmann et al. (2021)	https://github.com/PyPSA/atlite

### Resource availability

#### Lead contact

Further information and requests for resources and reagents should be directed to and will be fulfilled by the lead contact, Ebbe Kyhl Gøtske (ekg@mpe.au.dk).

#### Materials availability

This study did not generate new unique reagents.

### Method details

In the following, the full procedure to achieve the climate model projected inflow time series reported in this study is described. The time series are obtained using the location of the hydropower plants (JRC, 2019), the geometry of the upstream basins (Lehner and Grill, 2013), runoff data from regional climate models (Giorgi et al., 2009), and the open-source software Atlite (Hofmann et al., 2021). The methodology to obtain inflow time series was introduced by Liu et al. (2019) who obtained such time series of hourly temporal resolution for China using reanalysis weather data. The workflow in this study is illustrated in Figure 1, and this section will describe (in this order) how historical inflow observations are acquired, how and which climate models are acquired, how the upstream areas of the basins are determined, how runoff is aggregated on a country-level and converted into reservoir energy inflow, how the modeled reservoir energy inflow is calibrated with historical observations, and how the climate change effect is evaluated.

#### Historical inflow

The European Network of Transmission System Operators for Electricity (ENTSO-E, 2020) Transparency Platform provides hourly hydropower generation and weekly reservoir filling level for Austria, Bulgaria, France, Italy, Portugal, Romania, Switzerland, Montenegro, Norway, Spain, and Sweden with a period ranging from 2016 to 2019. Data from Holmqvist (2017), REE (2020), and Swedenergy (2020) provide a wider time range of historical data for Norway (1958–2017), Spain (1990–2019), and Sweden (1980–2019), which is used instead of ENTSO-E.

Using an energy balance, the weekly reservoir filling level can be expressed as:

(Equation 1) $$V_{w+1}=V_{w}+E_{w}-\sum_{h=1}^{168}G_{h}-E_{w}^{\text{loss}}\;,$$

where $V_{w}$ is the reservoir filling level at week *w*, $V_{w+1}$ the same at week w+1, $E_{w}$ is the energy inflow, $G_{h}$ is the electricity generation at hour *h*, and $E_{w}^{\text{loss}}$ is the energy loss. By collecting hourly electricity generation and weekly reservoir filling level at ENTSO-E and neglecting the energy loss, the weekly energy inflow $E_{w}$ is approximated with Equation 1. The validity of this approximation is evaluated for Norway and Spain by comparing the approximated inflow with historical inflow from Holmqvist (2017) and REE (2020). The comparison is illustrated in Figure S1 which shows good coherence.

#### Climate models

Climate model data is acquired from EURO-CORDEX (Giorgi et al., 2009; WCRP, 2020) driven by GCMs from the Coupled Model Intercomparison Project - Phase 5 (CMIP5) with $0.11^{\circ }\times 0.11^{\circ }$ (≈12km×12km) horizontal and daily resolution. Each climate model is given by a combination of a GCM and an RCM. An ensemble consisting of five different GCMs (MPI-M-MPI-ESM-LR (Giorgetta et al., 2013), ICHEC-EC-EARTH (Hazeleger et al., 2012),CNRM-CERFACS-CM5 (Voldoire et al., 2013), MOHC-HadGEM2-ES (Collins et al., 2011), and NCC-NorESM1-M(Bentsen et al., 2013)) and two RCMs (RCA4 (Samuelsson et al., 2011)and HIRHAM5 (Christensen et al., 2007)) is applied in this study. Each GCM-RCM combination is investigated at three different representative concentration pathways (RCPs), RCP2.6, RCP4.5, and RCP8.5 (Pachauri and Meyer, 2020). The 5 × 2 × 3 matrix of combinations is shown in Table S3. Although some climate models do not contain data for all RCPs, the three scenarios are well represented with a total of six models assuming RCP2.6, eight RCP4.5, and ten RCP8.5, which constitutes an ensemble of 24 climate models. As a preliminary step, the interannual, inter-RCM, inter-GCM, and climate change-induced variability of the runoff is evaluated for the RCP8.5 scenario.

As a frame of reference, we define the period from 1991 to 2020 as the beginning of the century (BOC) representing the historical climate, and from 2071 to 2100 as the end of the century (EOC) representing the future. The climate model runoff is indexed with the notation ${\bar{R}}_{ijkl}$ in which index *i* represents the considered period (i=1 for BOC and i=2 for EOC), *j* the GCM, *k* the RCM, and *l* the RCP. The bar indicates the mean annual value within a period of 30 years. Some climate models are subject to different calendar systems. E.g. NorESM1-M does not include leap years and HadGEM2-ES uses a 360 days per year calendar with 30 days in each month. Furthermore, HadGEM2-ES does not include the year 2100, and, thus, only 29 years will describe the EOC period with this GCM. In these irregular situations, the missing days will be filled out by repeating the data of the previous day, month or year.

#### Upstream area determination

Surface runoff is the excess water from precipitation, i.e. rain and snow, or meltwater from snow or glaciers that does not enter the soil (infiltration) or evaporates into the atmosphere. The runoff movement is actuated by gravity, and the flow direction is determined by the topography, i.e. the shape of the land surface. The runoff will eventually reach a river, a lake, a pond etc. and be collected in here. A drainage basin (referred to as *basin*) is the area of land that drains all flow of water, e.g. runoff, river streams, groundwater etc., to a common outlet, e.g. a river, reservoir, the ocean etc (USGS, 2020). The basins can be delineated from topographic maps, using the Pfafstetter coding scheme from Verdin and Verdin (1999). The Pfafstetter code itself indicates the number of subdivisions and the basin type. If the last digit is odd the basin is an *interbassin* meaning that the main stem of the river runs through it. If the last digit is even, the basin is a *tributary basin* and only has tributaries connecting to the river. Finally, if the last digit is zero, it is an isolated *internal basin* that does not have any river connections with surrounding basins. Increasing the level, increases the resolution, thus the size of each basin is reduced. By this approach, one digit is added to the Pfafstetter code, e.g. a level 1 code has one digit, level 2 has two digits, etc. The amount of runoff that is caught by the river does not only depend on the runoff in one single subbasin, but does, due to the interconnection, also depend on the runoff collected by the river-connecting basins. The basin area that contributes to the runoff in a reservoir, is termed as the *upstream area*.

Figure S3 considers one particular power station located in basin 2510407, in Southern Norway. The boundaries of the watersheds, i.e. the basins delineations, are collected from the HydroBASINS with Pfafstetter level 6,7, and 8. Locations of the hydropower plants are collected from the JRC Hydropower Database. See Figure S2 for a map of the installed hydropower capacities in Europe. From the basin delineation level 7, it is shown that basin 2510408 (tributary) and 2510409 (interbasin) contribute to the flow in basin 2510407, and they all constitute the upstream area for the reservoirs in this specific basin. As Figure S3 also shows, the resolution of the delineation is refined with Pfafstetter level 8. Liu et al. (2019) applied level 7 to identify the upstream areas at a $0.3^{\circ }\times 0.3^{\circ }$ runoff grid. In this work, the runoff data is acquired at a $0.11^{\circ }\times 0.11^{\circ }$. In line with this refinement, we determine the upstream basins based on a level 8 basins delineation. A preliminary test, see Figure S4, shows that in 15 countries, level 8 leads to a higher correlation between modeled and historical inflow.

#### Runoff aggregation

Using the distance between the runoff grid cell and the reservoir, and assuming a flow speed of 1 m/s, the runoff is first aggregated in the basins connected to a reservoir, followed by a country-level aggregation and conversion into a volumetric flow rate. Country-aggregated volumetric inflow is calculated with Equation 2:

(Equation 2) $$Q_{c}=\sum_{b}^{M}\frac{R_{bc}A_{b}}{\text{Δ}t}\;,$$

where *b* is the upstream basin index, *M* is the number of upstream basins within country *c*, $R_{bc}$ is the aggregated runoff in basin *b* and country *c* [m], $A_{b}$ is the surface area of the basin $\left[{\text{m}}^{2}\right]$, Δt is the temporal resolution of the runoff data [s], and $Q_{c}$ is the country-aggregated volumetric inflow $\left[{\text{m}}^{3}/\text{s}\right]$.

#### Energy conversion

The aggregated volumetric inflow can be transformed into units of energy to make it comparable with historical inflow. We consider one grid cell *i* containing runoff at the altitude $z_{i}$ which eventually reaches a hydropower reservoir.

**Figure:**
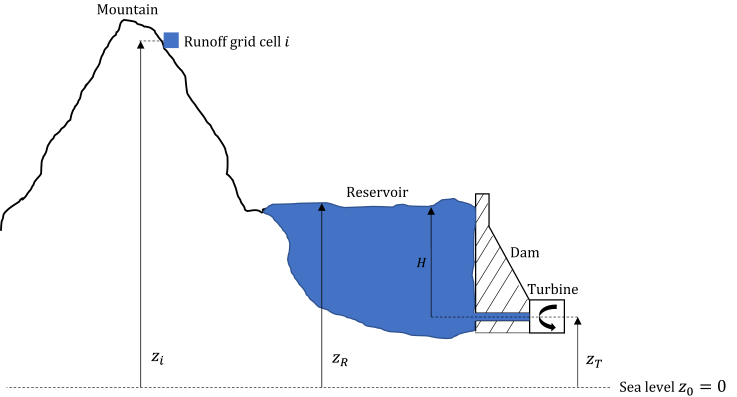

Assuming that the initial velocity of the runoff is zero, i.e. negligible kinetic energy, the maximum theoretically extractable energy $E^{\text{max}}$ (per time step) of the runoff at altitude $z_{i}$ by the turbine at altitude $z_{T}$ is estimated with Equation 3:

(Equation 3) $$E^{\text{max}}=\eta \rho gQ\left(z_{i}-z_{T}\right)\text{Δ}t\;,$$

where η is the turbine efficiency which is assumed to be 1, *g* is the gravitational acceleration ($9.81\text{m}/{\text{s}}^{2}$), ρ is the water density ($1000\text{kg}/{\text{m}}^{3}$), and *Q* is the flow rate of the runoff. In reality, some of the potential energy of the water in the grid cell is converted into kinetic energy when moving down the terrain and subsequently lost when reaching the reservoir; thus, the available energy $E^{\text{available}}$ is reduced to:

(Equation 4) $$E^{\text{available}}=\rho gQ\left(z_{R}-z_{T}\right)\text{Δ}t=\rho gQH\text{Δ}t\;,$$

where $z_{R}$ is the altitude of the hydropower reservoir and *H* is the (hydraulic) head. Note that for run-of-river power plants the kinetic energy would not be lost, since this type of plant extracts the kinetic energy directly.

In Equation 2, we rewrite $R_{bc}=\sum_{i}^{N}R_{ibc}$, where *N* is the number of grid cells within one basin. Furthermore, we approximate the head in Equation 4 by altitude $z_{i}$, due to the lack of data on reservoir head of every existing hydropower plant, which yields:

(Equation 5) $$E_{c}^{\mathrm{mod}el}=\rho g\sum_{b}^{M}\sum_{i}^{N}z_{i}R_{ibc}A_{b},$$

where $E_{c}^{\text{model}}$ is the country-aggregated modeled energy inflow. The head approximation leads to the error $e_{z}$ caused by the mismatch between the altitude $z_{i}$ and the reservoir head *H*:

(Equation 6) $$e_{z}=z_{i}-H$$

The bias caused by the mismatch will be adjusted in the calibration.

#### Calibration

In the following, we introduce a calibration factor which we will refer to as the retain factor (RF). It quantifies the level of water lost due to evaporation, transpiration, irrigation, or groundwater infiltration before reaching the reservoir, which all contribute to an energy loss $E^{\text{loss}}$. The retain factor is determined by the ratio between the seasonal inflow obtained with the climate models at the beginning of the century (1991–2020) and the historical observations (1991–2019):

(Equation 7) $$RF_{cm}=\frac{Observed\;\inf\;low}{Modelled\;\inf\;low\;at\;BOC}=\frac{E_{cm}^{hist}}{E_{cm}^{\mathrm{mod}el}},$$

where index *c* indicates country (1–22) and *m* month of an average year (1–12).

According to the definition presented in Equation 7, RF corresponds to the fraction of upstream-runoff caught by the hydropower reservoirs, i.e. the *retained* water. The retain factor includes the bias correction for the mismatch between the head and the runoff altitude, $\bar{e_{z}}$ (Equation 6). Evaporation taking place while water is stored in the reservoir can also impact the potential power generation (Turner et al., 2017). The retain factor also accounts for this. The obtained monthly retain factors ($RF_{c,m}$) for Norway and Spain, used in the calibration of climate model data, are shown in the figure below, where (a) shows the comparison of the modeled (right axis), prior to calibration, average monthly inflow at BOC period (1991–2020) and observed (left axis) inflow (1991–2019), and (b) the corresponding retain factors estimated according to Equation 7.

The model performance is, prior to calibration with the retain factor, evaluated on the Pearson correlation between modeled and observed inflow:

(Equation 8) $$r_{\hat{E}E}=\frac{\sum_{m=1}^{12}\left({\hat{E}}_{m}-\langle \hat{E}\rangle \right)\left(E_{m}-\langle E\rangle \right)}{\sqrt{\sum_{m=1}^{12}\left({\hat{E}}_{m}-\langle \hat{E}\rangle^{2}\right)}\sqrt{\sum_{m=1}^{12}{\left(E_{m}-\langle E\rangle \right)}^{2}}}\;,$$

where ${\hat{E}}_{t}$ is the modeled inflow at month *m*, $\left\langle \hat{E}\right\rangle$ is the modeled mean inflow, $E_{m}$ is the observed inflow at month *m*, and ⟨E⟩ is the observation mean inflow.

The comparison between the climate model obtained inflow with the observed, for every country, is shown in Figures S5 and S6, which indicate the Pearson correlation as well. Values below 0.3 are considered weak, between 0.3 and 0.7 moderate, and above 0.7 strong correlations (Ratner, 2009). The comparison shows a strong (positive) correlation between the modeled and observed mean monthly inflow for 14 countries. Countries in the Balkan Peninsula and north of it show moderate correlations. Later, this evaluation is extended by conducting a cross-validation of the calibrated model.

**Figure:**
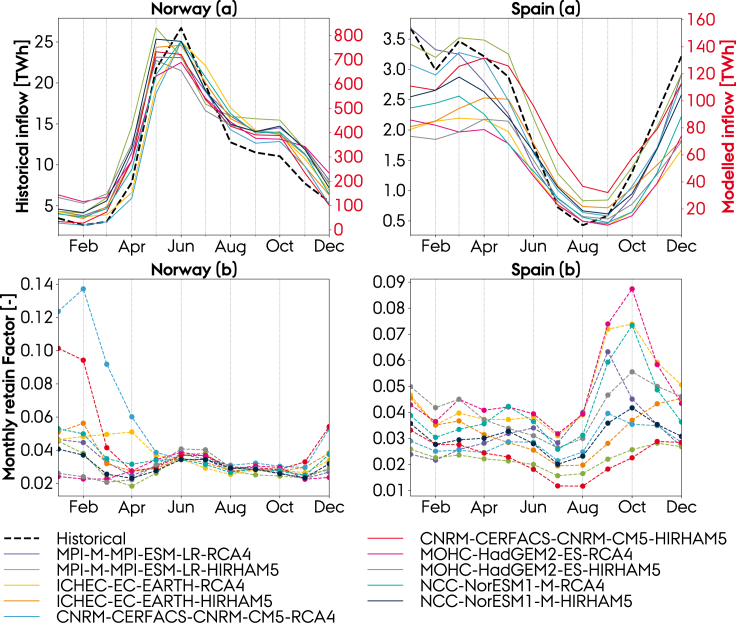

The modeled inflow for both BOC and EOC is calibrated with the retain factor:

(Equation 9) $$E_{cm}^{\text{calibrated}}=RF_{cm}\left(E^{\text{loss}},\bar{e_{z}}\right)E_{cm}^{\mathrm{mod}\text{el}}=RF_{cm}\left(E^{\text{loss}},\bar{e_{z}}\right)\rho g\sum_{b}^{M}\sum_{i}^{N}z_{i}R_{ibc}A_{b}$$

By using Equation 9 for the calibration of inflow at EOC, we assume that climate change does not affect the seasonality and magnitude of the retain factor. In reality, the global temperature rise is accompanied by a larger evaporation rate, which increases the energy loss. This is not accounted for in our approach.

#### Model cross-validation

With the described methodology and given data, it is here investigated how well and how accurate the predictions of reservoir inflow represent the actual natural hydrological characteristics. For countries with abundant historical data, i.e. Norway, Spain, and Sweden, we split the data into intervals of four years, which equals the time span of the observations collected from ENTSO-E. In this way, we will see how the different weather years affect the result, i.e. we will see the impact of e.g. training (determining the retain factor) with a set including a wet year versus one with a dry year.

The historical data ranges from 1991 to 2019. To simulate a climate projection, the time span is divided into two: A training period, in which the retain factor is determined, and a testing period, in which the calibrated model is applied to make a forecast. The training is based on data from 1991 to 2002 and testing on data from 2003 to 2019.

The accuracy of our model is estimated with the normalized root-mean-square-error (RMSE):

(Equation 10) $$\text{RMSE}=\frac{\sqrt{\frac{1}{12}\sum_{m=1}^{12}{\left({\hat{E}}_{m}-E_{m}^{\text{hist}}\right)}^{2}}}{\langle E_{m}^{\text{hist}}\rangle }\;,$$

where ${\hat{E}}_{m}$ is the monthly inflow predicted by the model and $E_{m}^{\text{hist}}$ is the observed monthly inflow. Furthermore, the Pearson correlation, indicates the similarity in the seasonal inflow from the projection and the historical mean observation. The two measures (root-mean-square error and Pearson correlation) will be evaluated for the train-test combinations listed in Table S2.

Again, we compare the mean inflow profiles, since we aim to estimate a representation of the climate and not of one particular year. The listed tests are performed for every climate model, leading to a total of 120 seasonal profiles, each representing a different set of training and testing weather data and a different climate model. The resulting forecasts for Norway within the period from 2003 to 2019 and the observed mean seasonal profile within the same period are shown in the figure below. Furthermore, the historical range (i.e. the range of the historical mean profiles within the three train and four test periods) is indicated.

**Figure:**
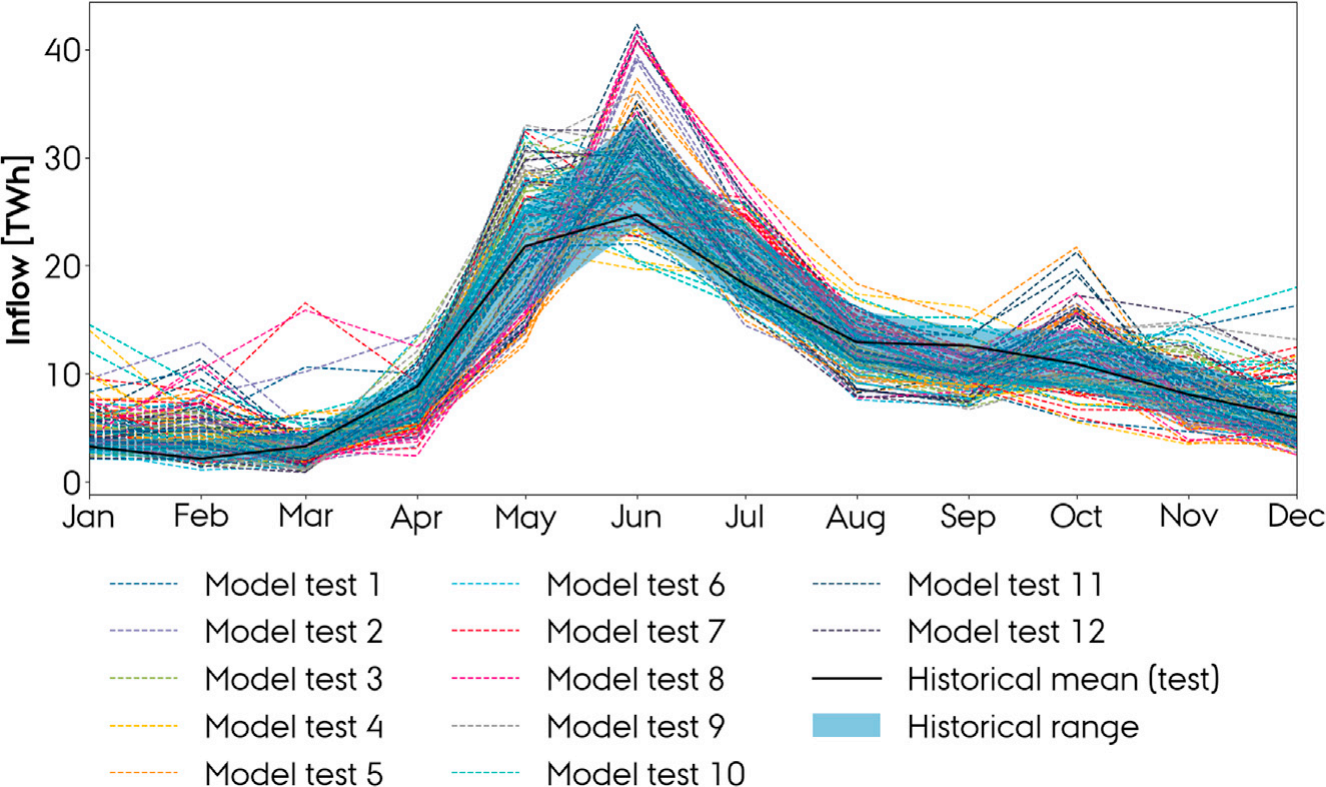

First, it shows that the seasonality in inflow is well resembled by the model. Second, it shows a high variation in the results depending on which weather period is used for the training or testing. The historical range does, however, reveal that inflow is subject to a great natural interannual variability. In June, which is the month with highest inflow, inflow has historically varied within a range of approximately 10 TWh, which corresponds to approximately 7.5% of the total annual inflow in Norway. On the other hand, it also shows that the result is highly influenced by which climate model is used, which is clearly shown for e.g. “Model test 7″ which has a range of almost 20 TWh in June due to different signals from the climate models. For Spain and Sweden, see Figure S7.

Table S2 lists the accuracies (RMSE) and Pearson correlations for the different tests and for the historical observations, relative to the historical mean. It shows that the correlations remain strong for Norway, Spain, and Sweden regardless of the considered weather period. The accuracy is, however, influenced. For Norway, it ranges from 25% in the most regular weather period to 42% in the most irregular one. In average, this corresponds to a climate model deviation (RMSE = 32%), to the historical mean, which is larger than the historical (maximum) natural variability. For Sweden (RMSE = 34%), this is also the case, whereas the average deviation for Spain (RMSE = 43%) yields a similar magnitude as the historical.

From the table, it occurs that the period from 1995 to 1998 was the most irregular one for all three countries since it historically showed the highest deviation from (and lowest correlation with) the 30-year mean (26.7%, 43.2%, and 24.8% for Norway, Spain, and Sweden). This is also shown in Figure S8 which presents the seasonal profiles at the various historical eras.

The mean accuracies achieved in the forecasts across all twelve tests are:

(Equation 11) $$\begin{array}{ll} \left\langle {\text{RMSE}}^{\text{Norway}}\right\rangle & =32.0\text{\%}\\ \left\langle {\text{RMSE}}^{\text{Spain}}\right\rangle & =42.7\text{\%}\\ \left\langle {\text{RMSE}}^{\text{Sweden}}\right\rangle & =34.3\text{\%} \end{array}$$

For Norway and Sweden, this corresponds to 19.8% and 38% larger deviations from the historical mean compared to the observations in the most irregular period. For Spain, the root-mean-square-error yields a similar magnitude as the most irregular period.

#### Climate change effect

The climate change effect *S* is determined by comparing the runoff from EOC with BOC. E.g. the absolute change in mean daily runoff when assuming RCP8.5 is calculated as:

(Equation 12) $$S_{jk}^{R}={\bar{R}}_{2jk3}-{\bar{R}}_{1jk3}$$

The climate change effect on the mean annual inflow is evaluated by calculating the relative change:

(Equation 13) $$S_{jk}^{E}=\frac{{\bar{E}}_{2jk3}-{\bar{E}}_{1jk3}}{{\bar{E}}_{1jk3}}\times 100$$

### Quantification and statistical analysis

A statistical analysis is made, to evaluate the significance of the changes in inflow registered by the climate models, by performing a paired t test (Kim, 2015). One of the underlying assumptions of this hypothesis test, is that the data follows a normal distribution. To test this assumption, a Shapiro-Wilk test (Shapiro and Wilk, 1965)is performed. In addition, the distributions are represented with probability density functions estimated with the kernel density estimation from Seaborn (Waskom, 2021). In the t test, we test if the null hypothesis, stating that the difference in mean annual inflow at the two eras is zero, can be rejected. In both the normality (Shapiro-Wilk) and t test, we apply a significance level of 0.05.

## Data Availability

Historical and climate model projected inflow time series have been deposited at Zenodo: 5106349 and are publicly available as of the date of publication. DOIs are listed in the key resources table. The historical inflow input data for countries such as Bosnia-Herzegovina, Croatia, Czech Republic, Finland, Germany, Poland, Serbia, Slovakia, Slovenia, Hungary, and North Macedonia reported in this study cannot be deposited in a public repository for the sake of the data provider. To request access, contact the source listed in the key resources table. All original code has been deposited at Zenodo: 5109492 and is publicly available as of the date of publication. DOIs are listed in the key resources table. Any additional information required to reanalyze the data reported in this paper is available from the lead contact upon request.
