# Supplementary material for: Future operation of hydropower in Europe under high renewable penetration and climate change
Source: iScience. 2021 Aug 19;24(9):102999. doi: 10.1016/j.isci.2021.102999 (PMC8413898; doi:10.1016/j.isci.2021.102999)
Supplement: Document S1. Figures S1–S19 and Tables S1 and S2 [file mmc1.pdf]

iScience, Volume *24*

Supplemental information

Future operation of hydropower in Europe under high renewable penetration and climate change

Ebbe Kyhl Gøtske and Marta Victoria

Table S1. Combinations of general circulation models (GCMs), regional climate models (RCMs), and representative concentration pathways (RCPs) considered in this study, Related to STAR Methods

GCM / RCM		**MPI-ESM-LR** (Giorgetta et al., 2013)	**EC-EARTH** (Hazelheger, 2012)	**CNRM-CM5** (Voldoire, 2013)	**HadGEM2-ES** (Collins et al., 2011)	**NorESM1-M** (Bentsen et al., 2013)
**RCA4**	RCP2.6	x	x		x	x
(Samuelsson et al., 2011)	RCP4.5	x	x	x	x	x
	RCP8.5	x	x	x	x	x
**HIRHAM5**	RCP2.6		x		x	
(Christensen et al., 2007)	RCP4.5		x		x	x
	RCP8.5	x	x	x	x	x

Table S2. Model cross-validation results, Related to Star Methods
The table presents the root-mean-square-errors and Pearson correlations for model test 1, 2, ..., 12, relative to the historical observations. As a comparison, the range of root-mean-square-errors of the observed inflow relative to the historical mean, to represent the natural variability between the four-year periods (N), is presented.

No.	Train, Test	Norway RMSE, r	Spain RMSE, r	Sweden RMSE, r
1	1991-1994, 2003-2006	$\pm 25.2\%$, 0.94	$\pm 35.5\%$, 0.86	$\pm 25.1\%$, 0.94
2	1995-1998, 2003-2006	$\pm 40.2\%$, 0.88	$\pm 56.1\%$, 0.76	$\pm 36.9\%$, 0.91
3	1999-2002, 2003-2006	$\pm 28.2\%$, 0.95	$\pm 34.9\%$, 0.84	$\pm 30.3\%$, 0.98
4	1991-1994, 2007-2010	$\pm 24.6\%$, 0.93	$\pm 35.8\%$, 0.85	$\pm 30.0\%$, 0.92
5	1995-1998, 2007-2010	$\pm 37.0\%$, 0.88	$\pm 60.3\%$, 0.77	$\pm 39.5\%$, 0.89
6	1999-2002, 2007-2010	$\pm 25.9\%$, 0.95	$\pm 35.1\%$, 0.84	$\pm 31.5\%$, 0.97
7	1991-1994, 2011-2014	$\pm 29.9\%$, 0.92	$\pm 36.6\%$, 0.85	$\pm 33.1\%$, 0.91
8	1995-1998, 2011-2014	$\pm 42.3\%$, 0.87	$\pm 53.1\%$, 0.74	$\pm 45.8\%$, 0.89
9	1999-2002, 2011-2014	$\pm 31.2\%$, 0.95	$\pm 38.0\%$, 0.80	$\pm 38.9\%$, 0.97
10	1991-1994, 2015-2019	$\pm 28.8\%$, 0.92	$\pm 37.8\%$, 0.86	$\pm 31.4\%$, 0.92
11	1995-1998, 2015-2019	$\pm 39.3\%$, 0.88	$\pm 54.1\%$, 0.76	$\pm 36.7\%$, 0.91
12	1999-2002, 2015-2019	$\pm 31.1\%$, 0.94	$\pm 34.8\%$, 0.84	$\pm 32.0\%$, 0.97
N	1991 - 1994	$\pm 15.2\%$	$\pm 31.7\%$	$\pm 15.8\%$
N	1995 - 1998	$\pm 26.7\%$	$\pm 43.2\%$	$\pm 24.8\%$
N	1999 - 2002	$\pm 14.4\%$	$\pm 20.3\%$	$\pm 13.4\%$
N	2003 - 2006	$\pm 17.5\%$	$\pm 22.3\%$	$\pm 15.2\%$
N	2007 - 2010	$\pm 9.7\%$	$\pm 16.7\%$	$\pm 13.6\%$
N	2011 - 2014	$\pm 10.9\%$	$\pm 29.8\%$	$\pm 11.1\%$
N	2015 - 2019	$\pm 16.7\%$	$\pm 24.0\%$	$\pm 9.8\%$

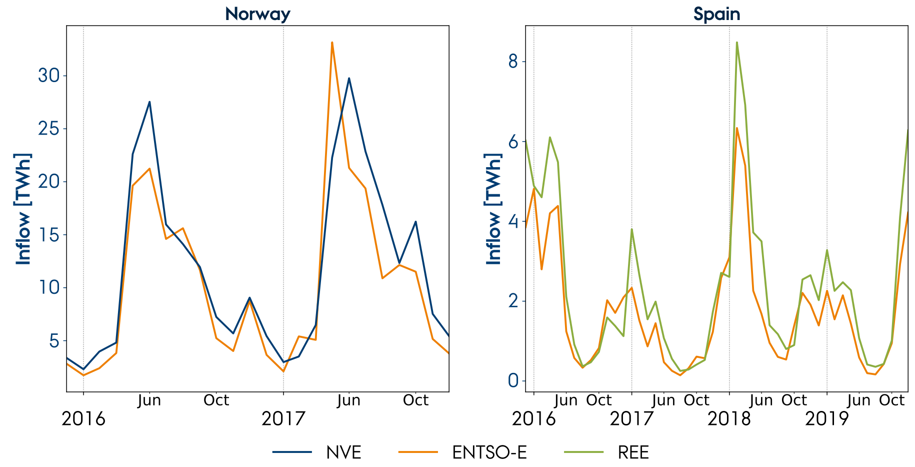

Figure S1: **Inflow approximated from electricity generation and reservoir filling level data from ENTSO-E compared with historical inflow from NVE (Norway) and REE (Spain), Related to STAR Methods**

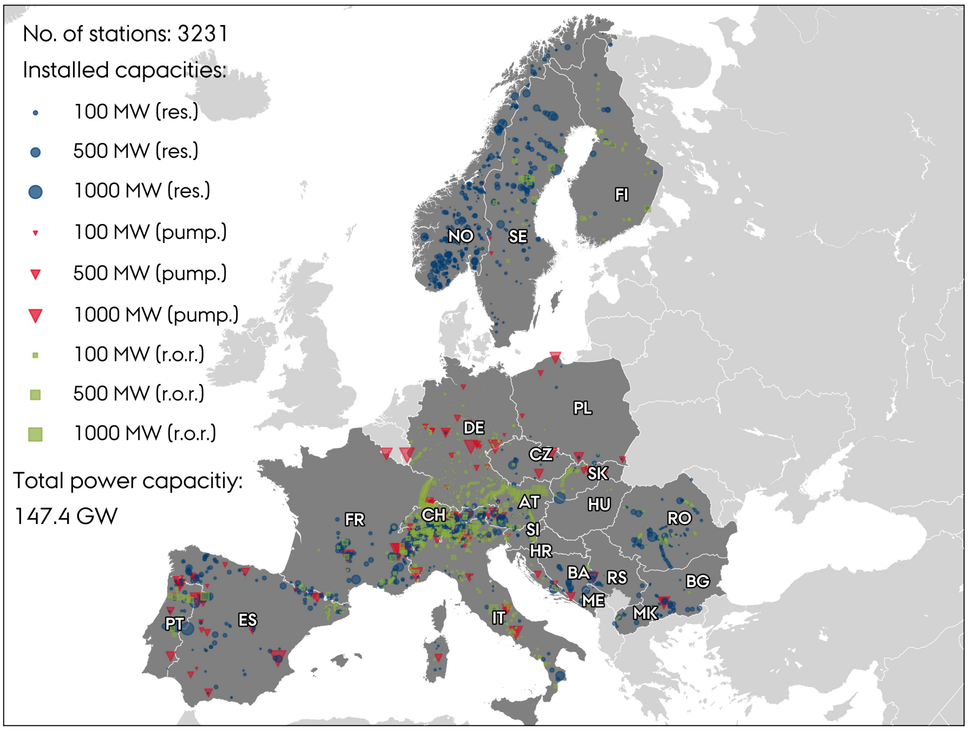

Figure S2: **Location and power capacities of reservoirs (res.), pumped-hydro storage facilities (pump.), and run-of-river (r.o.r) power plants from JRC, Related to STAR Methods**

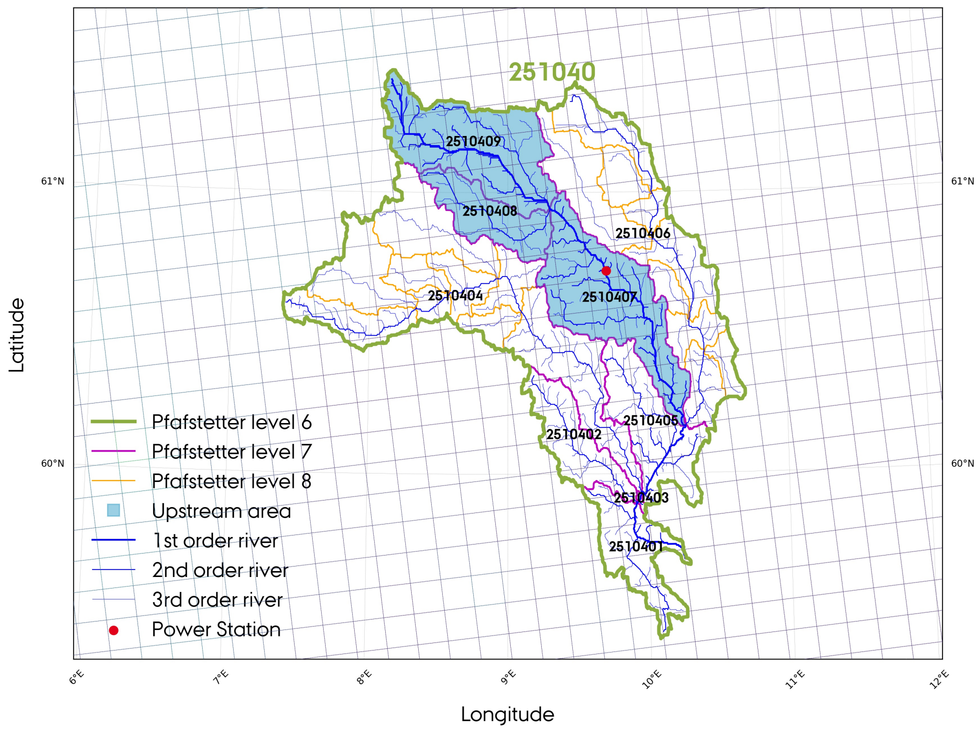

Figure S3: **Upstream area depiction of one power plant in Norway, using a level 7 basin delineation, with an overlay of the EURO-CORDEX EUR-11 grid, Related to STAR Methods**

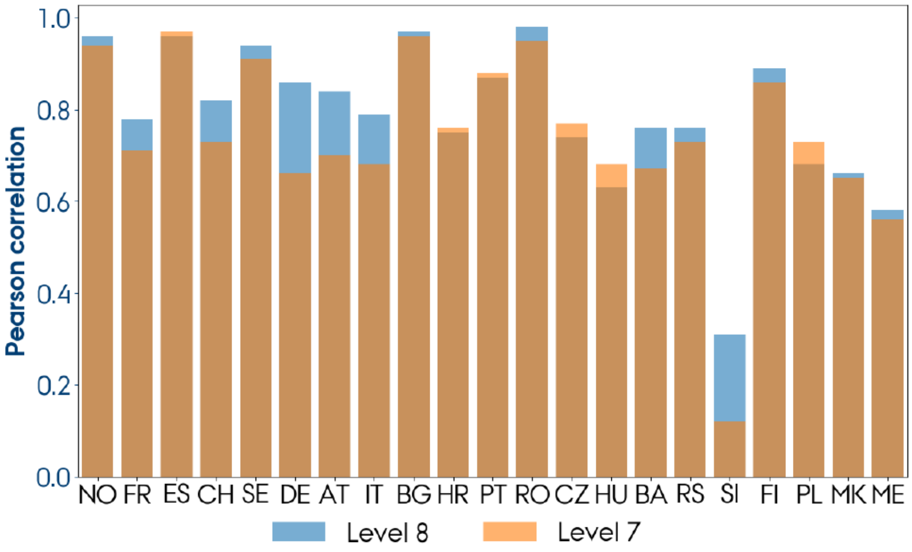

Figure S4: **Pearson correlation between modelled and historical inflow at Pfafstetter level 7 and 8 basins delineation, Related to STAR Methods**
Modelled inflow is based on the single GCM-RCM combination MPI-ESM-LR-RCA4. Slovakia is excluded from this figure since it is negative in both cases, see Supplemental Figure S5.

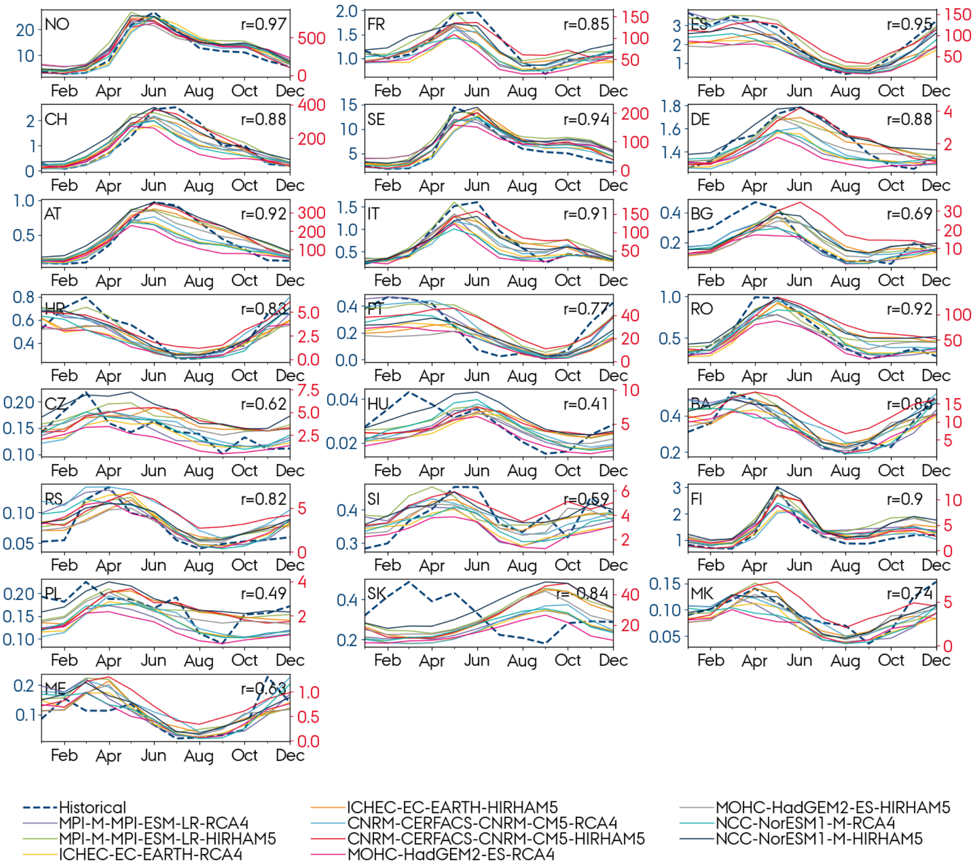

Figure S5: **Comparison of modelled, prior to calibration, and observed seasonal inflow for 22 European countries, Related to STAR Methods**

The figure shows modelled (right axis) and observed (left axis) seasonal inflow in TWh for the 22 European countries. The variable r indicates the mean Pearson correlation obtained for the 10 (different GCM-RCM combinations) climate models. The modelled seasonal inflow for Slovakia does not show coherence with the historical observations, illustrated by the climate models consistently predicting peak inflow during fall when it historically has occured during spring, leading to a negative Pearson correlation.

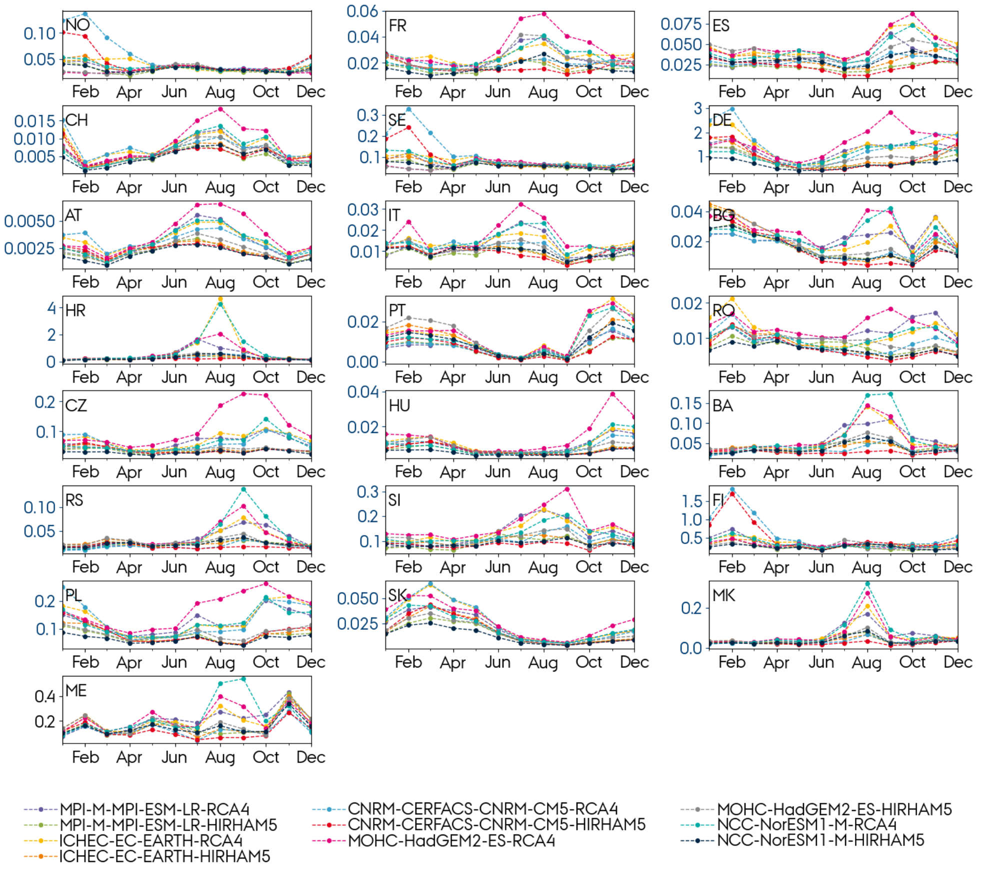

Figure S6: **Obtained retain factors for 22 European countries, Related to STAR Methods**
The retain factors are obtained with the 10 different GCM-RCM combinations.

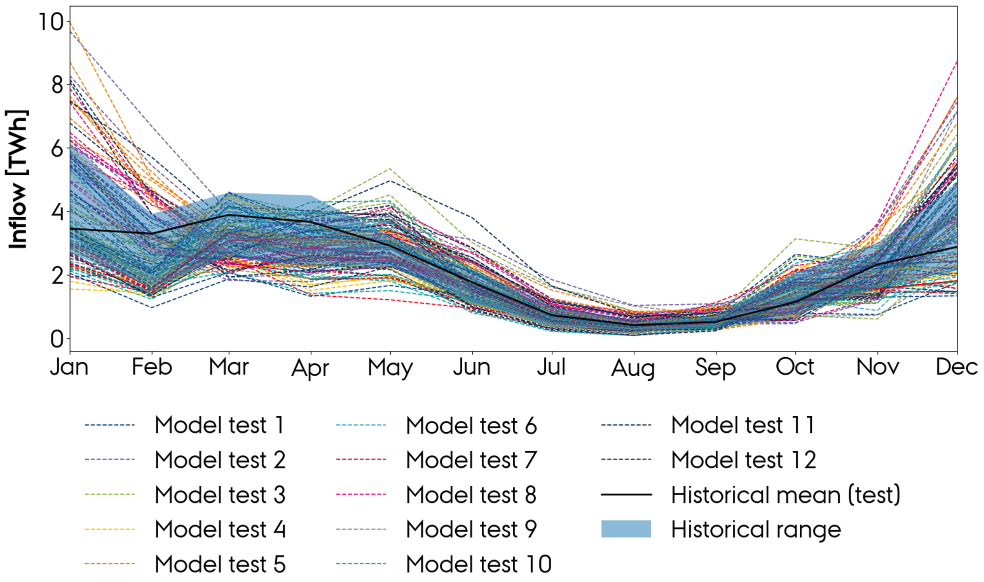

(a)

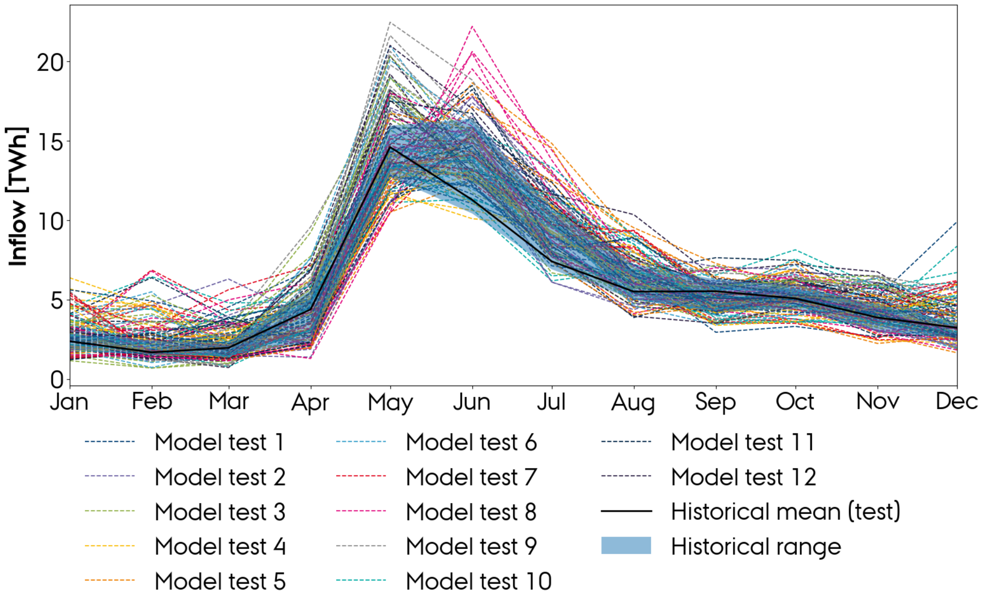

(b)

Figure S7: **Model forecasts, following the calibration with the retain factor, and the observed mean inflow within the test period for (a) Spain and (b) Sweden, Related to STAR Methods**

The dashed lines with different colors represent different training or testing years, see Supplemental Table S2. As a comparison, the historical range (blue shade) within the entire time span (i.e. from 1991 to 2019) is illustrated.

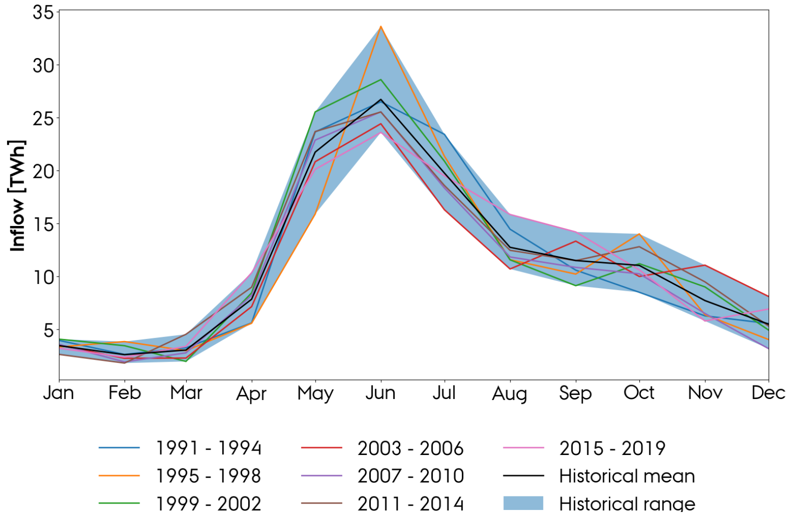

(a)

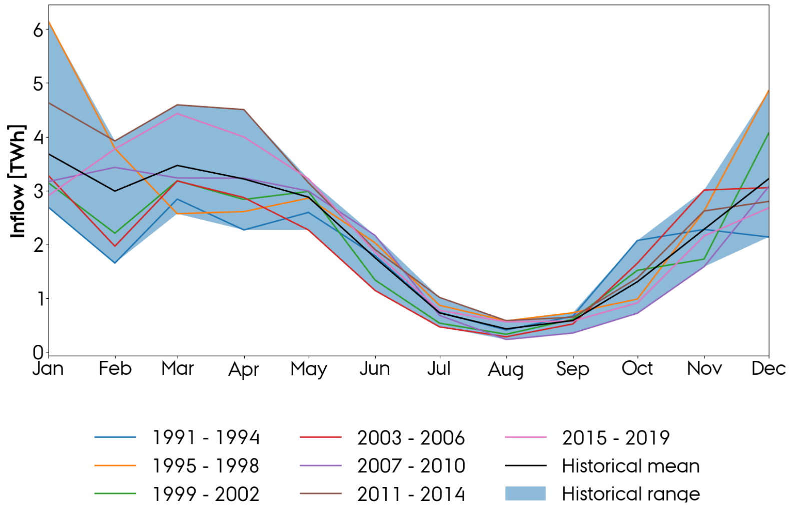

(b)

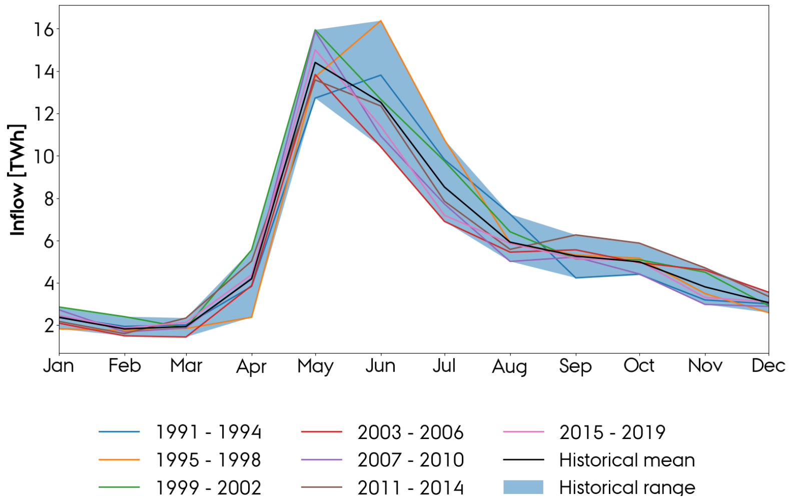

(c)

Figure S8: Historical variations in the inflow for (a) Norway, (b) Spain, and (c) Sweden, Related to STAR Methods

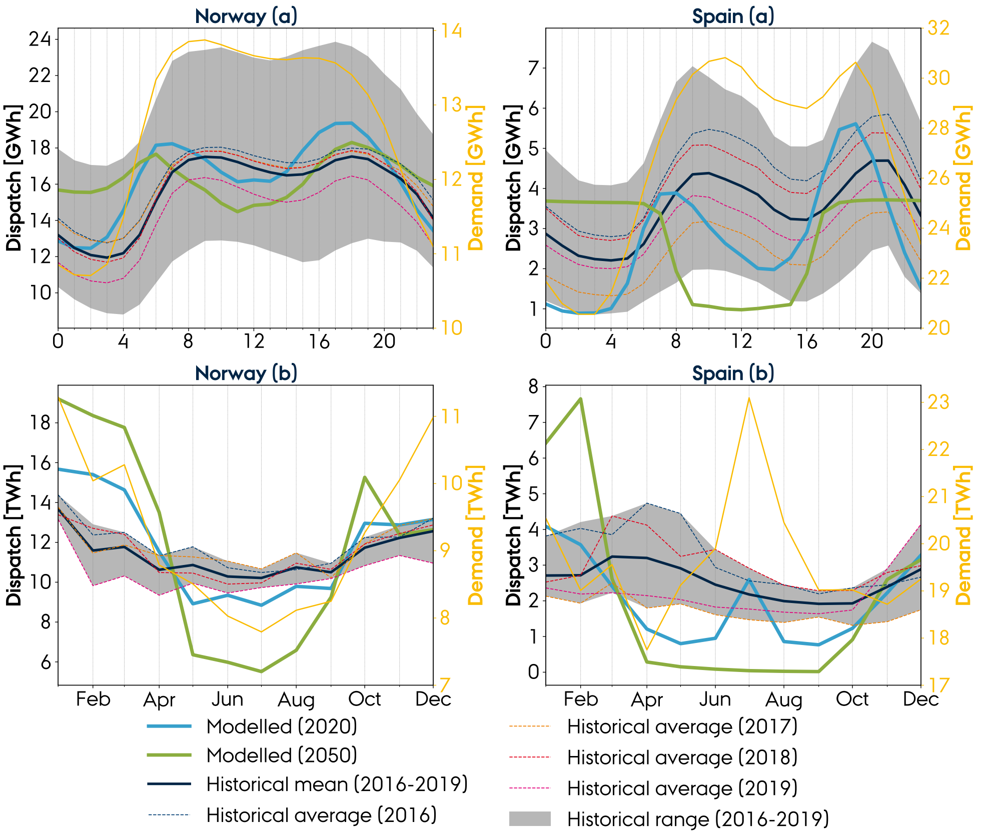

Figure S9: **Modelled and historical (a) intraday and (b) seasonal operation of hydropower plants, Related to Figure 2**

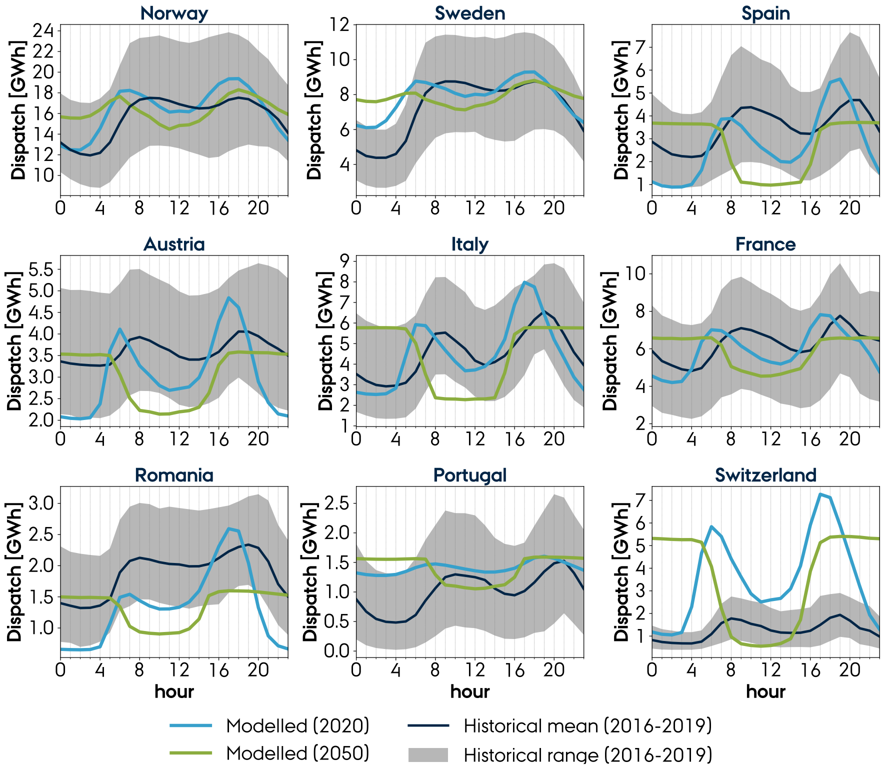

(a)

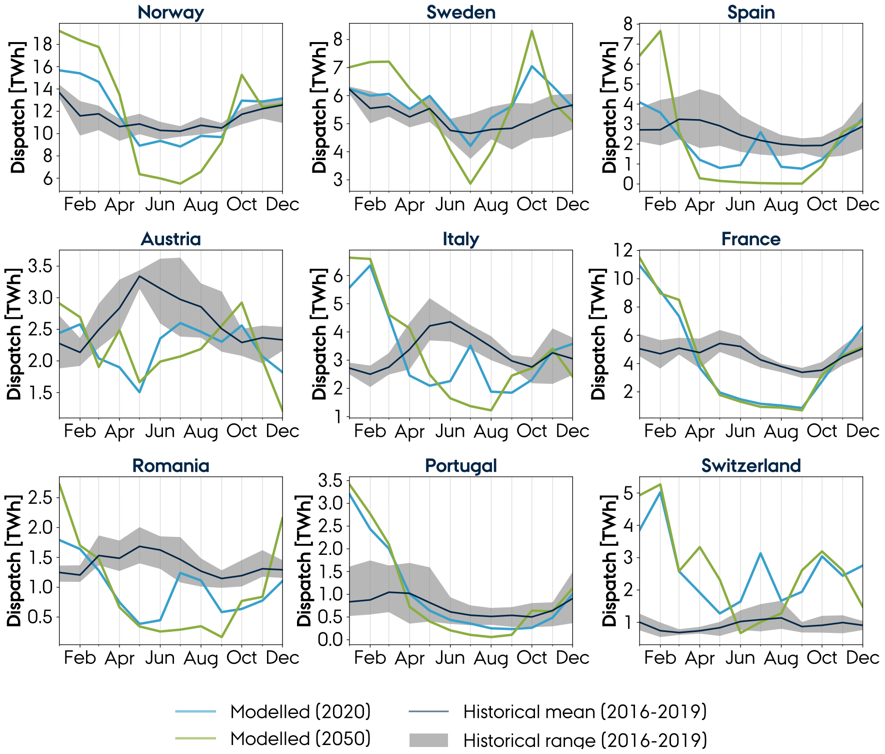

(b)

Figure S10: **Modelled and historical (a) intraday and (b) seasonal operation of hydropower plants, Related to Figure 2**
The historical observations are based on hourly reservoir and run-of-river hydropower generation from ENTSO-E.

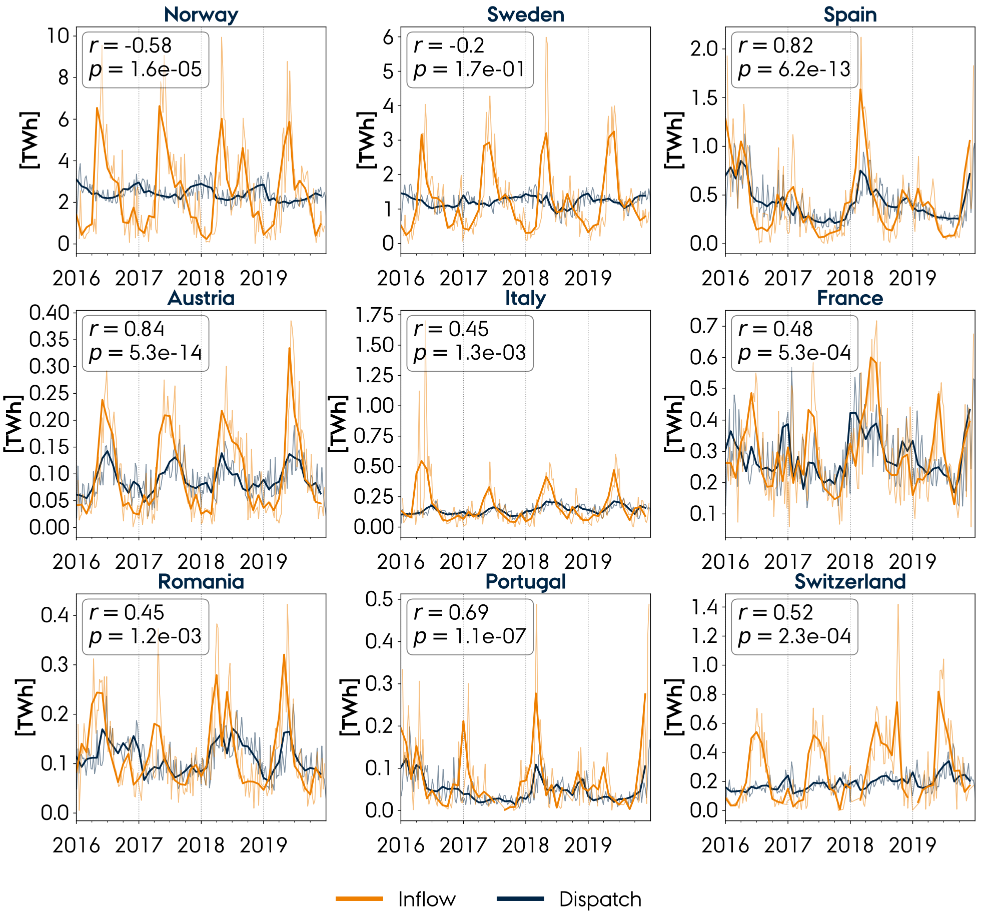

(a)

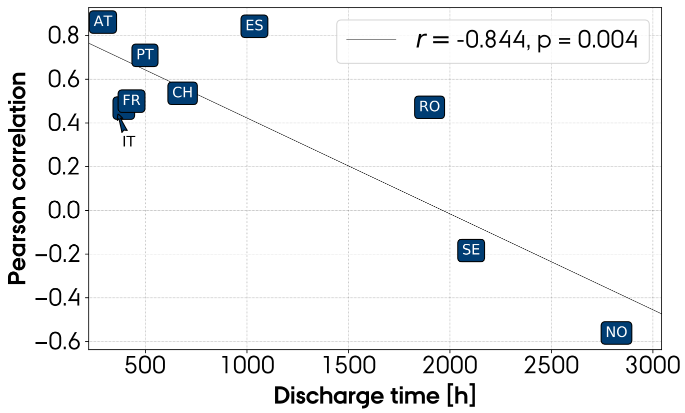

(b)

Figure S11: **Historical inflow and dispatch correlations, Related to Figure 3**
The figure shows (a) historical hydropower reservoir inflow and dispatch from 2016 to 2019 from ENTSO-E (thin lines indicate weekly values and thick monthly averaged), and (b) inflow-dispatch correlation and discharge time. In (a) Pearson correlation r represents the correlation between inflow and dispatch, and in (b) r represents the correlation between the inflow-dispatch correlation and the discharge time. Furthermore, p is the statistical significance of the Pearson correlation.

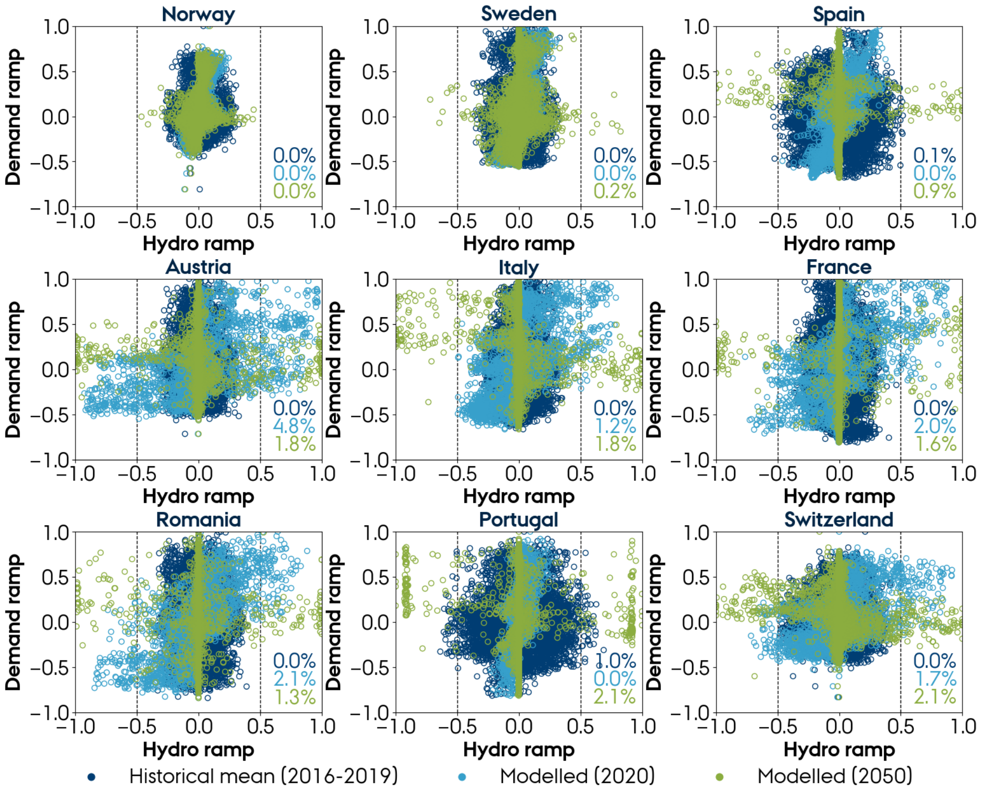

Figure S12: **Scatter plot of hourly hydropower ramp rates, Related to Figure 2**
The figure shows hydropower ramp rates (x-axis) at given electricity demand ramp rates (y-axis) for the modelled 2020 (cyan), 2050 (green), and historical (dark blue) power production. The percentage of hours in a year at which the absolute value of the ramp rates are above 0.5 is indicated for the three data sets. Historical observations are based on hourly reservoir dispatch from ENTSO-E.

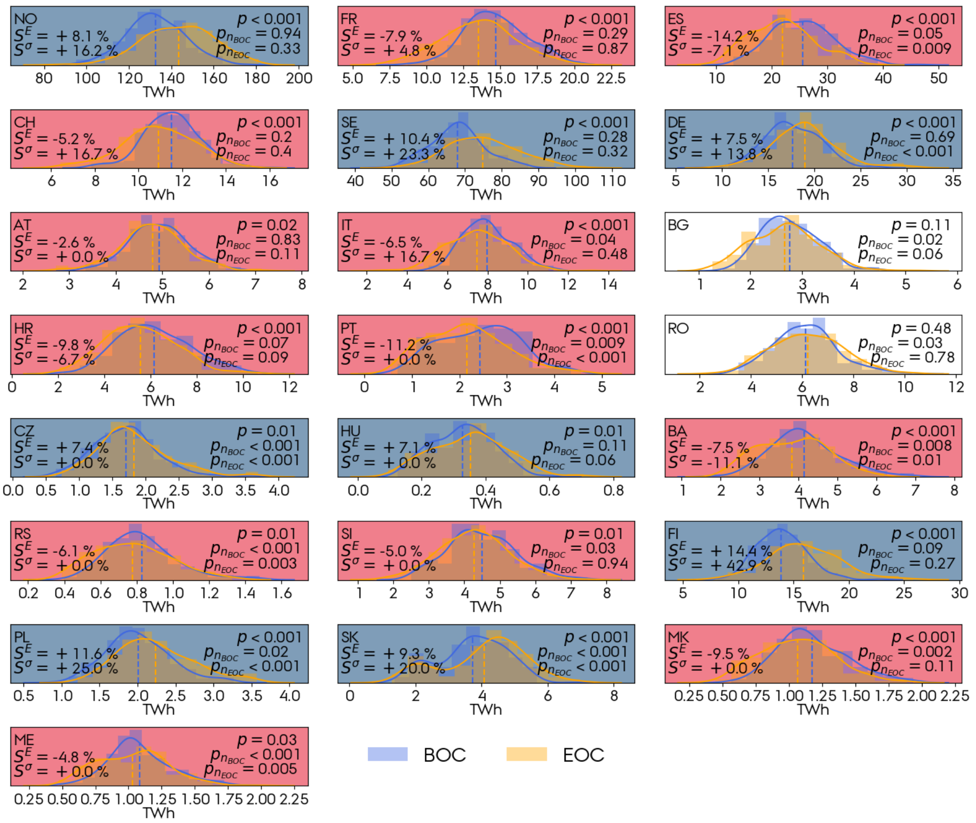

Figure S13: **Ensemble distributions of the annual inflow at the BOC and EOC 30-years periods for the RCP4.5 scenario, Related to Figure 7**

Dashed lines indicate the mean values of the distributions. The sets are normally distributed if $p_n > 0.05$ based on a Shapiro-Wilk test. For the countries with a statistically significant change ($p < 0.05$), a blue (red) shade indicates an increase (decrease) in the annual inflow. S^E and S^σ correspond to the relative change in annual inflow and interannual variability caused by climate change.

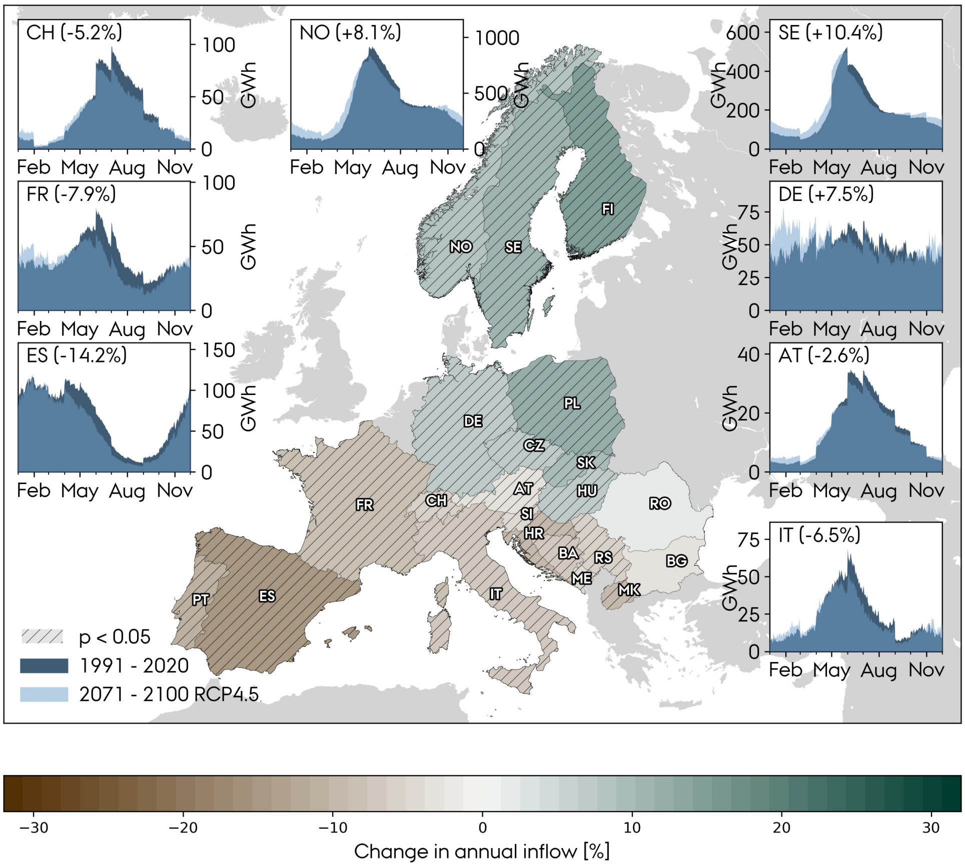

Figure S14: **Ensemble mean relative change in annual inflow and change in seasonal inflow profile for RCP4.5, Related to Figure 8**
Dashed patterns indicate results that are statistically significant ($p<0.05$)

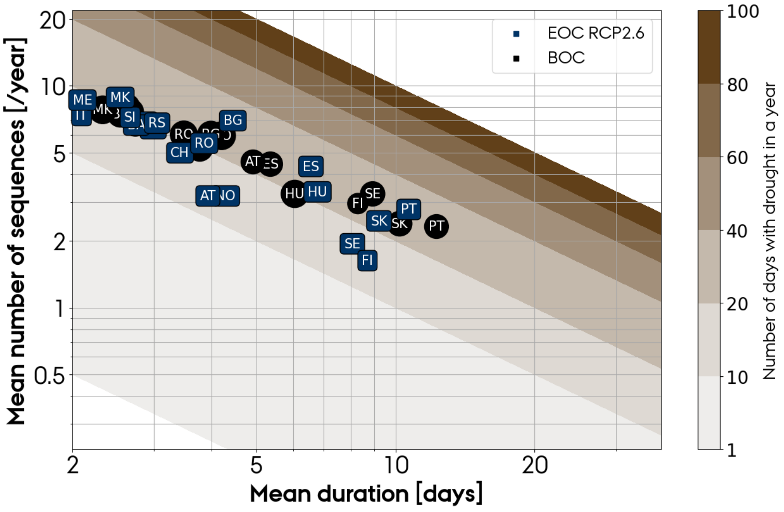

(a)

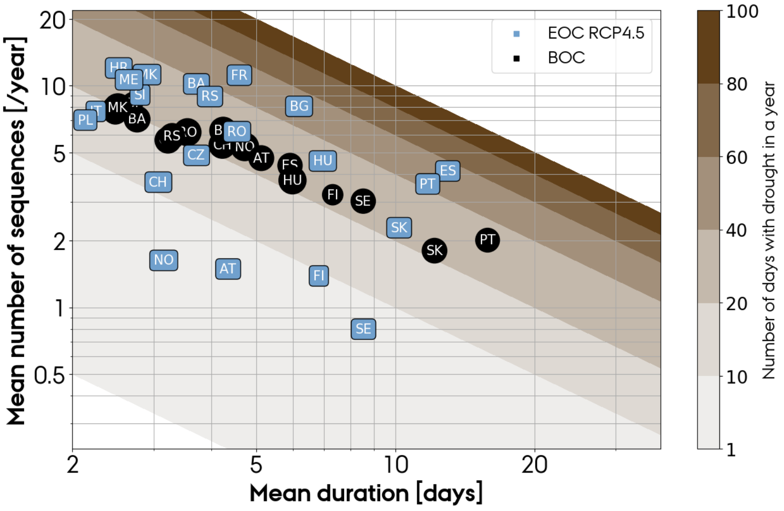

(b)

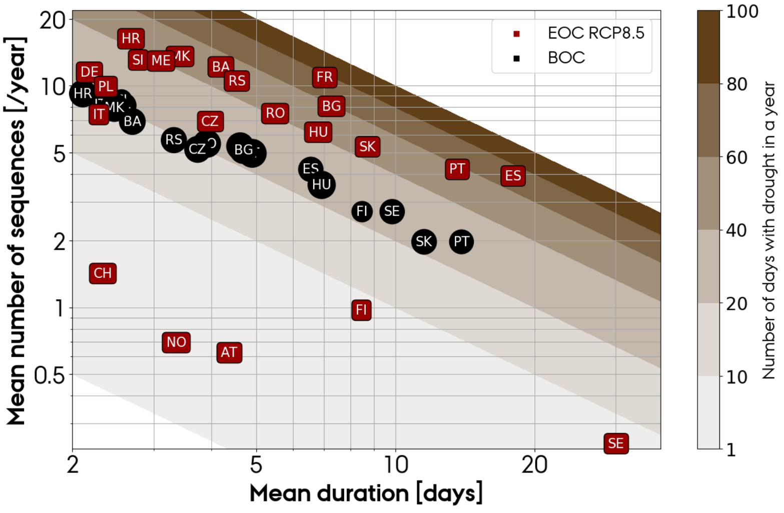

(c)

Figure S15: **Duration and frequency of drought periods, Related to Figure 10**
Drought periods are determined as consecutive days with inflow less than the 10th percentile of the BOC period, evaluated for the BOC and EOC periods at the (a) RCP2.6, (b) RCP4.5, and (c) RCP8.5 scenario.

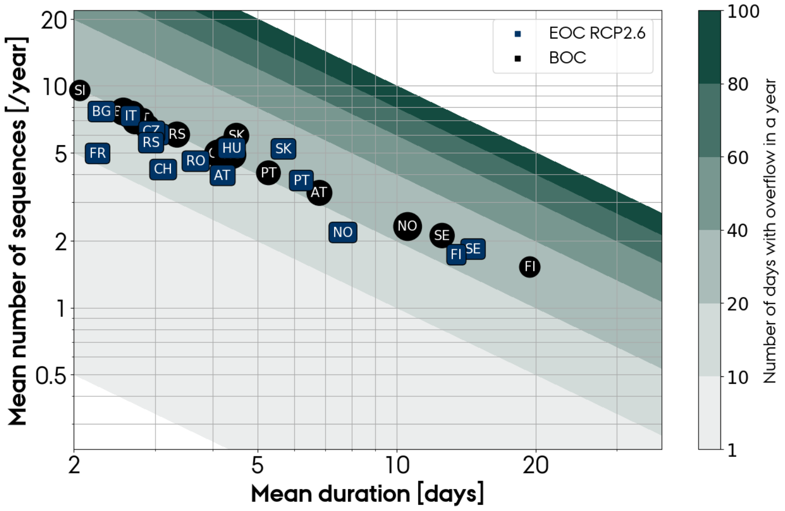

(a)

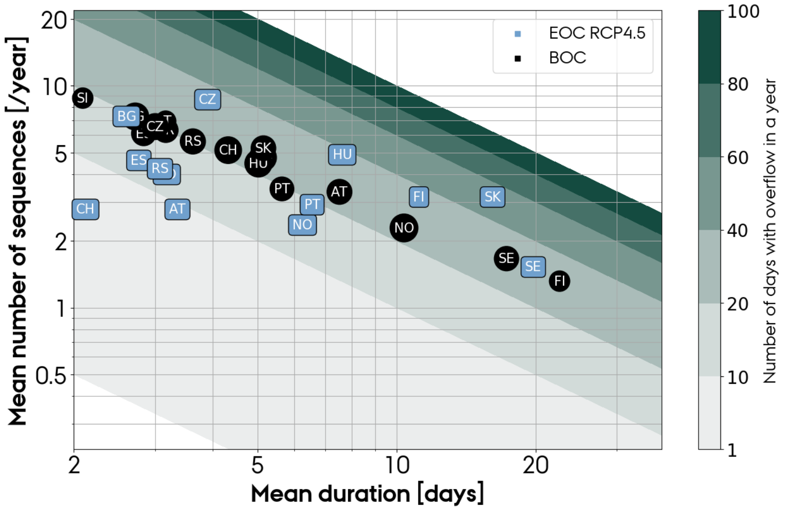

(b)

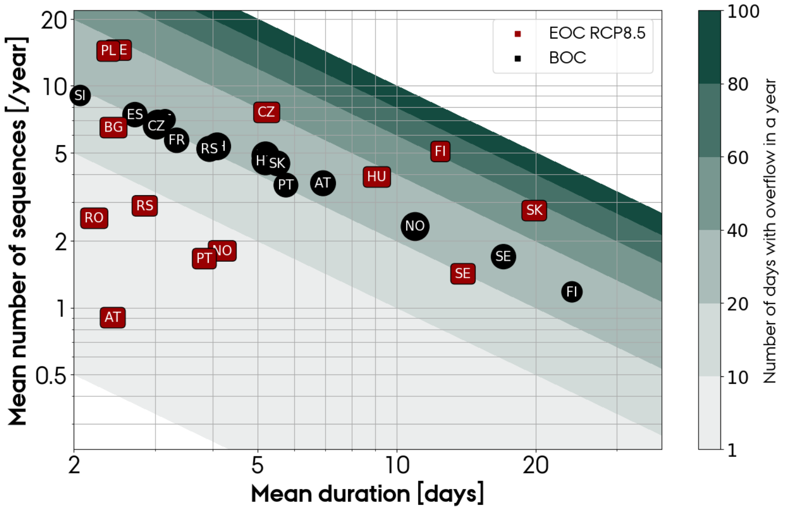

(c)

Figure S16: **Duration and frequency of overflow periods, Related to Figure 10**
Overflow periods are determined as consecutive days with inflow larger than the 90th percentile of the BOC period, evaluated for the BOC and EOC periods at the (a) RCP2.6, (b) RCP4.5, and (c) RCP8.5 scenario.

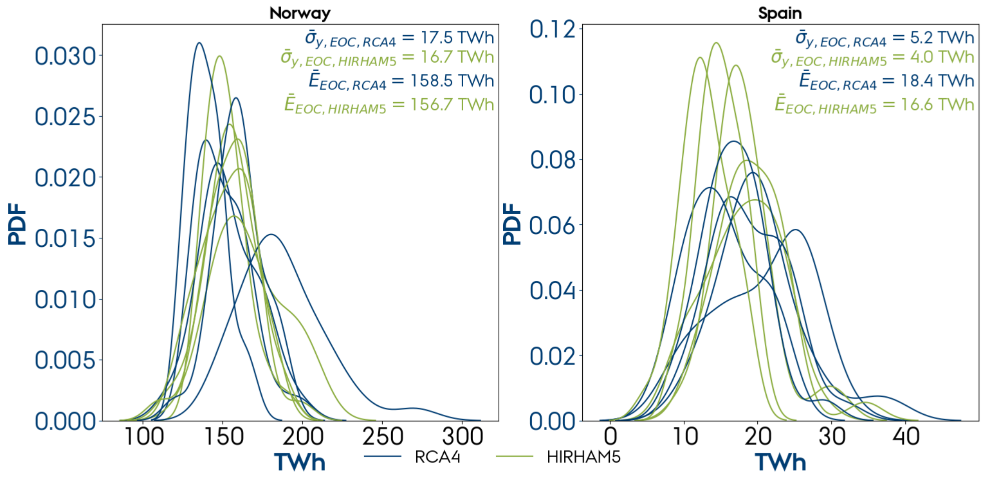

(a)

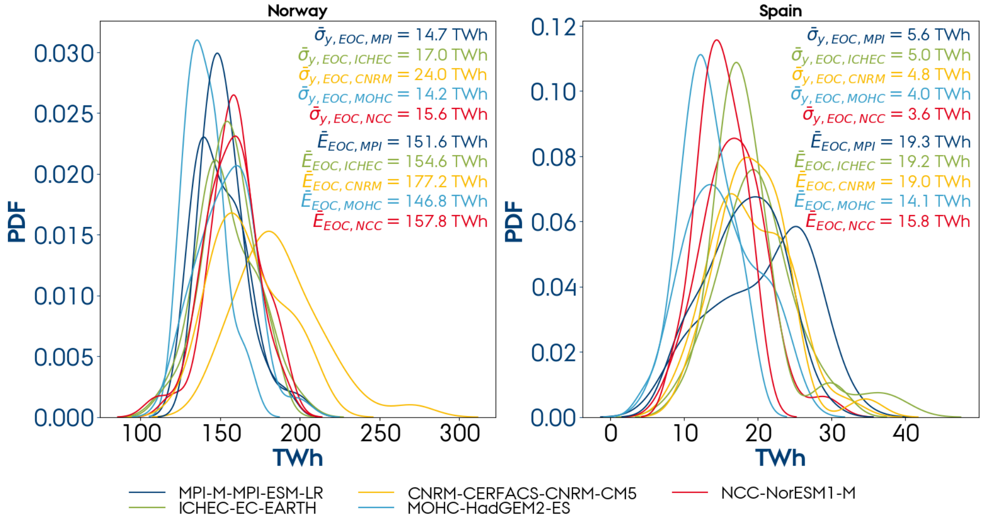

(b)

Figure S17: **Comparison of inter-RCM and inter-GCM variability, Related to Figure 6**
The figure shows (a) inter-RCM and (b) inter-GCM variability of the annual inflow at the end of the century obtained with the 10 climate models. $\bar{\sigma}_y$ is the mean interannual variability (standard deviation) and $\bar{E}$ is the mean annual inflow.

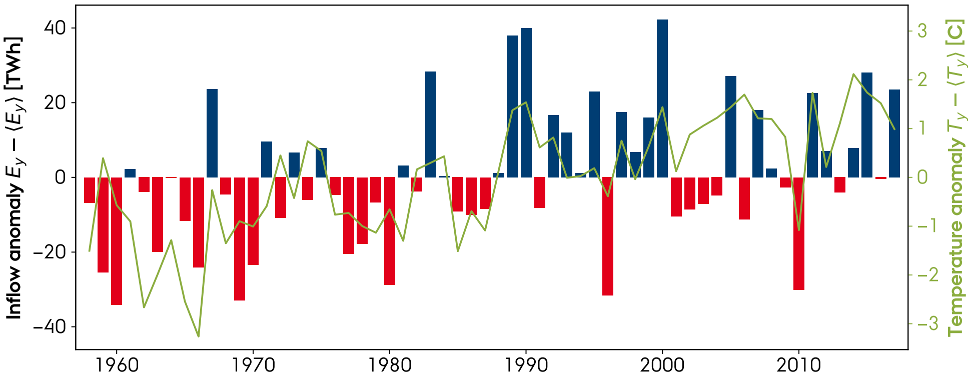

Figure S18: **Annual inflow relative to historical mean in Norway, Related to Figure 4**
The figure shows annual inflow relative to historical mean in Norway from 1958 to 2017 (left axis) from Holmqvist (2017). The red (blue) bar indicates a year with inflow less (larger) than the historical mean, and the green line plot indicates the air temperature collected from Hersbech et al. (2018) relative to the historical mean (right axis).

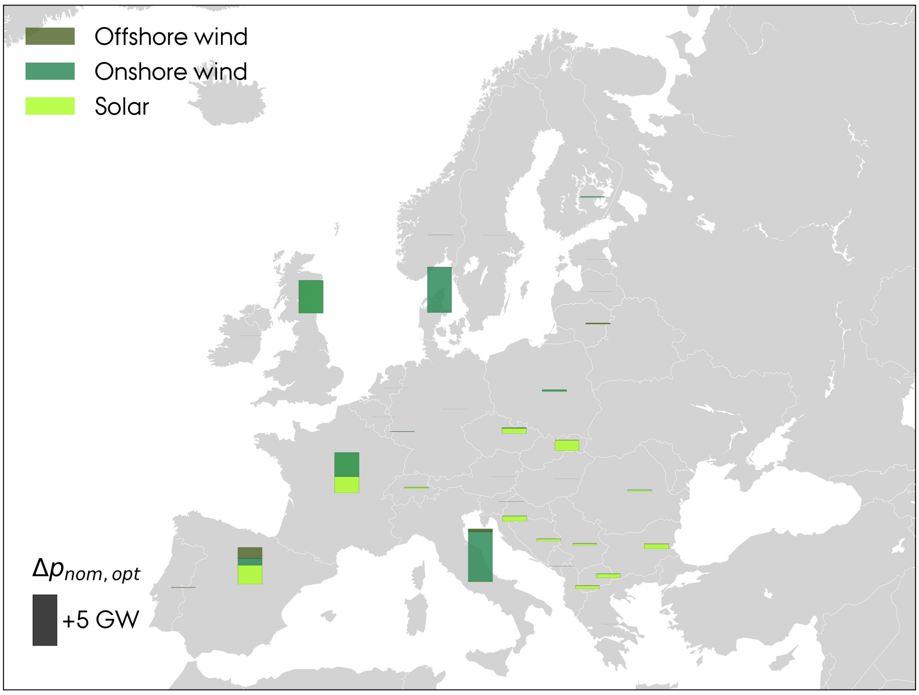

Figure S19: **Changes in the optimal wind and solar power capacities due to the climate change effect on hydropower resources, Related to Figure 8**
